# From endodormancy to ecodormancy: the transcriptional landscape of apple floral buds

**DOI:** 10.3389/fpls.2023.1194244

**Published:** 2023-07-14

**Authors:** Sangeeta Sapkota, Mohamed Salem, Khalil R. Jahed, Timothy S. Artlip, Sherif M. Sherif

**Affiliations:** ^1^ Virginia Agricultural Research and Extension Center, Virginia Tech, Winchester, VA, United States; ^2^ Department of Horticulture, Michigan State University, East Lansing, MI, United States; ^3^ Department of Statistics, Virginia Tech, Blacksburg, VA, United States; ^4^ Appalachian Fruit Research Station, United States Department of Agriculture – Agricultural Research Service, Kearneysville, WV, United States

**Keywords:** bud dormancy, transcriptomics, jasmonic acid, flowering time, endodormancy, ecodormancy

## Abstract

This study endeavors to explore the transcriptomic profiles of two apple cultivars, namely, ‘Honeycrisp’ and ‘Cripps Pink,’ which represent late and early-blooming cultivars, respectively. Using RNA-sequencing technology, we analyzed floral bud samples collected at five distinct time intervals during both endodormancy and ecodormancy. To evaluate the transcriptomic profiles of the 30 sequenced samples, we conducted principal component analysis (PCA). PC1 explained 43% of the variance, separating endodormancy and ecodormancy periods, while PC2 explained 16% of the variance, separating the two cultivars. The number of differentially expressed genes (DEGs) increased with endodormancy progression and remained elevated during ecodormancy. The majority of DEGs were unique to a particular time point, with only a few overlapping among or between the time points. This highlights the temporal specificity of gene expression during the dormancy transition and emphasizes the importance of sampling at multiple time points to capture the complete transcriptomic dynamics of this intricate process. We identified a total of 4204 upregulated and 7817 downregulated DEGs in the comparison of endodormancy and ecodormancy, regardless of cultivar, and 2135 upregulated and 2413 downregulated DEGs in the comparison of ‘Honeycrisp’ versus ‘Cripps Pink,’ regardless of dormancy stage. Furthermore, we conducted a co-expression network analysis to gain insight into the coordinated gene expression profiles across different time points, dormancy stages, and cultivars. This analysis revealed the most significant module (ME 14), correlated with 1000 GDH and consisting of 1162 genes. The expression of the genes within this module was lower in ‘Honeycrisp’ than in ‘Cripps Pink.’ The top 20 DEGs identified in ME 14 were primarily related to jasmonic acid biosynthesis and signaling, lipid metabolism, oxidation-reduction, and transmembrane transport activity. This suggests a plausible role for these pathways in governing bud dormancy and flowering time in apple.

## Introduction

The dormancy of buds in temperate perennials constitutes a crucial adaptive mechanism for safeguarding reproductive tissues and buds from low winter temperatures. This phenomenon plays a significant role in determining the annual cycle of the deciduous fruit trees, including regrowth, flowering, and fruit production. The induction and release of dormancy can be triggered by shifts in temperature and photoperiod (Cooke et al., 2012), or temperature alone in species such as pear and apple (Heide and Prestrud, 2005). Winter dormancy in buds can be divided into two phases: endodormancy and ecodormancy (Lang, 1987). Endodormancy is an internally regulated process, where growth is suppressed even in favorable environmental conditions, while ecodormancy occurs as a result of unfavorable external conditions that prevent bud regrowth in spring. To transition from endodormancy, buds must be exposed to low temperatures, known as the chilling requirements (CR). Similarly, buds in ecodormancy require a specific amount of heat, known as the heat requirement (HR), to ensure proper budburst and flowering (Gibson and Reighard, 2002). CR and HR are specific to the species and genotype, and their fulfillment drives the transition of dormancy stages through the integration of multiple signaling pathways (Fadón et al., 2020).

Gaining a comprehensive understanding of dormancy and its regulation is of utmost importance in ensuring the sustainability of fruit production. This is particularly relevant in the face of global climate change, which has the potential to significantly alter the dormancy-regrowth cycle. For instance, unseasonably warm winter temperatures can impede the successful completion of the cold requirement (CR), leading to poor and inconsistent bud break and bloom (Rai et al., 2015). In temperate regions, rising temperatures may also cause an earlier onset of bud break and full bloom in spring, thereby increasing the likelihood of spring frost damage (Hoffmann and Rath, 2013; Stoeckli and Samietz, 2015). Studies have shown that the advancement of bud phenology and early bloom due to climate change increases the risk of frost damage, particularly in early-blooming apple cultivars, which may result in significant economic losses (Dalhaus et al., 2020). For example, the spring frost in Europe in 2017 resulted in a staggering loss of $3.3 billion for the fruit and wine grape industry (Lamichhane, 2021). Similarly, the spring frost observed in the eastern US in 2012 caused severe damage to half of the apple crop, incurring millions of dollars in losses as documented by Wolfe et al., 2018.

Several studies have delved into the genetic basis of dormancy in deciduous woody perennials (Liu and Sherif, 2019). Despite this, our comprehensive understanding of the underlying molecular and biochemical mechanisms involved in the regulation of dormancy and bloom time remains limited. In peach, a notable advancement in understanding the genetic control of dormancy was achieved through the identification of the evergreen (*evg*) mutant and subsequent mapping of the *evg* locus. The mapping revealed the presence of six tandemly arranged MADS-box genes, collectively referred to as the Dormancy Associated MADS-box (DAM) genes, which initiate endodormancy in peach (Bielenberg et al., 2008). Recent studies have confirmed the existence of DAM genes in pome fruits as well. For instance, Moser et al. (2020) showed that apple knockout mutants of the MdDAM1 genes were unable to enter dormancy and displayed constant growth. In pear, Tuan et al. (2017) demonstrated that DAM genes could be induced by low temperatures, via mediation by the transcription factor C-repeat binding factor (CBF). As transcription factors, the DAM genes may also regulate the expression of the FLOWERING LOCUS T (FT) gene, which induces flowering, and upregulate the expression of ABA biosynthesis genes in lateral flower buds (Tuan et al., 2017). During the progression of dormancy, the DAM genes can be suppressed epigenetically or degraded through small interference RNA or micro-RNA, facilitating endodormancy release (Leida et al., 2012; Niu et al., 2016; Rothkegel et al., 2017). These studies emphasize the crucial role of DAM genes in regulating the initiation and maintenance of endodormancy. However, it is likely that the physiological mechanisms, biochemical pathways, and gene networks associated with dormancy vary among species. Additionally, the studies conducted thus far have utilized a limited number of sampling points during the dormancy-regrowth cycle, thereby failing to capture the major cellular and molecular changes that occur during the transitions between dormancy stages.

The perception of environmental signals and the response to them can be influenced by various pathways, such as those mediated by hormones, reactive oxygen species (ROS), and carbohydrates, among others. These pathways can activate or suppress different genes (Olsen, 2006). Transcriptomics has been used to link gene expression profiles to the common molecular pathways involved in bud dormancy and the epigenetic mechanisms of dormancy regulation (Leida et al., 2010; Liu et al., 2012). However, the comprehensive mechanism of these pathways and their impact on dormancy regulation has yet to be fully understood (Leida et al., 2012). Studying the gene expression profile during the entire dormancy period based on chilling hours (CH) and growing degree hours (GDH) would provide valuable insight into the molecular events that regulate physiological processes. In this study, we are investigating the regulation of dormancy and bloom time in apples by using two distinct cultivars with different flowering dates - ‘Cripps Pink’ and ‘Honeycrisp’. The former is an early-bloom cultivar, while the latter is a late-bloom cultivar. Our goal is to identify genes that show different expression patterns between the two cultivars during different phases of dormancy. By doing so, we hope to gain insights into the pathways and gene networks that are linked to dormancy and bloom time in apples and other pome fruits. The utilization of apple genotypes with varying flowering dates presents a valuable opportunity to study the molecular aspects of dormancy and bloom time regulation. Such knowledge could be used to develop practical and effective management strategies to prevent potential frost injury and to breed climate-resilient cultivars in the future through marker-assisted selection.

## Materials and methods

### Plant material and RNA extraction

In our previous study (Sapkota et al., 2021a), we provided a detailed description of the experimental location and sampling. In summary, two apple cultivars, ‘Honeycrisp’ and ‘Cripps Pink’ with different bloom times were selected as previously illustrated (Sapkota et al., 2021a; Sapkota et al., 2021b). Floral buds were collected from the two apple cultivars at five different time points during endodormancy based on the accumulation of chilling hours (CH) (200, 400, 600, 800, and 1000 CH) and at three time points during ecodormancy based on the accumulation of growing degree hours (GDH) (1000, 2000, and 3000 GDH). We randomly selected three trees from each cultivar, each serving as a biological replicate for floral bud sampling. The buds were cryogenically ground into a fine powder using a Geno Grinder (SPEX SamplePrep, Metuchen, NJ, USA). Total RNA was extracted from the ground samples using the Cetyl Trimethyl Ammonium Bromide (CTAB) method (Wang and Stegemann, 2010), and the extracted RNA was treated with DNase and purified using an EZ RNA cleanup Kit (EZ Bioresearch LLC, Saint Louis, USA) according to the manufacturer’s instructions. The purified RNA, free of any DNA, was subjected to RNA-Seq analysis at five time points (200 CH, 600 CH, 1000 CH, 1000 GDH, and 3000 GDH), as well as qRT-PCR analyses at all time points. We selected five time points for RNA sequencing based on the onset, mid- and end-points of endodormancy, the initial stage of ecodormancy (1000 GDH), and the green tip stage (3000 GDH) in the early blooming cultivar.

### Library construction and sequencing

Library preparation and Illumina sequencing of the RNA samples were performed by the Novogene (Novogene, Sacramento, CA). Paired-end reads were generated from the libraries sequenced on an Illumina Hiseq 4000. RNA-seq yielded 40-50 million reads per library, which were evaluated for quality using FastQC (Andrews, 2010). Raw reads were then processed using Trimmomatic (Bolger et al., 2014) to remove adapters, unknown bases, and low-quality reads.

### Mapping and RNA-seq data analysis

The cleaned reads generated from the Trimmomatic were aligned to the apple reference genome (https://www.rosaceae.org/species/malus/malus_x_domestica/genome_GDDH13_v1.1) using HISAT2 (Kim et al., 2019). The read count was calculated using feature counts (Liao et al., 2014) and the number of fragments per kilobase of transcripts per million mapped reads (FPKM) of each gene was calculated as FPKM= 10^9*C/(N*L) where C is the number of fragments mapped to a gene, L is the gene length and N is the total mapped reads. Differentially expressed genes (DEGs) were determined using the DESEq2 package in R (Love et al., 2014) with the cut off value of |log2 fold change| > 1 and the adjusted p-value < 0.05 using Benjamini and Hochberg’s approach. The expression profile of all DEGs were clustered using pheatmap function under DESEq2 package. In addition, functional enrichment analysis was performed on DEGs using Gene Ontology (GO), and Kyoto Encyclopedia of Genes using clusterProfiler package in R (version 4.3.0) (Yu et al., 2012). Enriched GO terms and pathways were identified using significant DEGs with the statistical options based on hypergeometric statistical model and p-values adjusted to Hochberg method. The GO terms with p-adjusted ≤ 0.05 were considered to be significantly enriched.

### Differential Co-expression and network enrichment analysis

Identification of modules with highly correlated genes based on the DESEq2 normalized gene expression data was performed using Weighted Gene Correlation Network Analysis (WGCNA)- R package (Zhang and Horvath, 2005). The expression profile of the genes was hierarchically clustered into modules using a Pearson’s Correlation module eigengene (ME). Genes sharing a similar expression pattern form a co-expression module. Expression analysis of the genes within the module was performed using the R package “pheatmap”. GO network enrichment was analyzed using Arabidopsis homologs in Cytoscape using BiNGO (Maere et al., 2005).

### Reverse transcription quantitative PCR

Total RNA was purified and treated with DNase I to remove genomic DNA (gDNA), using RNA Clean & Concentrator – 5 kit (Zymo Research CORP., Irvine, CA, USA), following the manufacturer’s protocol. Applied biosystems High-Capacity cDNA Reverse Transcription Kit (Applied Biosystems, Waltham, MA, USA) was used for cDNA synthesis following the manufacturer’s instructions. The cDNA was diluted 10-fold for quantitative reverse transcription-polymerase chain reaction (qRT-PCR) analysis. Gene specific primers were designed using Primers3Plus software (Untergasser et al., 2007), and listed in **Table S1**. The specificity of the primer pairs was verified by melting curve analysis at the end of the RT-PCR. All primer pairs used in this study displayed a distinct, single peak in the melting curve analysis. Quantitative RT-PCR analyses were performed on the CFX Connect Real-Time PCR Detection System (Bio-Rad Laboratories, Inc., Hercules, CA, USA). The qRT-PCR reactions were done in 10 μL/reaction, using the Fast EvaGreen^®^ qPCR Master Mix (Biotium, Fremont, CA, USA). Reaction parameters were set to standard cycling mode as: 90°C for 5 min; 95°C for 20 sec; followed by 60°C for 20 sect (40 cycles). The relative normalized expression of each gene was analyzed using CFX manager software (Bio-Rad). Gene expression was normalized using two reference genes, *MdActin* (Zhou et al., 2017) and *MdGAPDH* (Malladi and Hirst, 2010), and then calculated relative to the expression level in the control sample (‘Cripps Pink’ at 200 CH). The expression of each gene was compared using a student’s t-test between ‘Honeycrisp’ and ‘Cripps Pink’ at each time point, using R programming language for statistical computing and graphics version 4.2.2. To validate the RNA-Seq results, the expression values obtained from qRT-PCR were correlated to FPKM values from RNA-Seq for each gene (**Table S3**).

## Results

### Transcriptomic profile clustered by dormancy stages and genotypes

‘Honeycrisp’ is a relatively late bloom cultivar; flowering a week later than ‘Cripps Pink’. Our previous investigations indicated that the two cultivars have similar chilling requirements of 1000 CH, and the difference in bloom time mainly arose from the differences in heat requirements between these two cultivars (Sapkota et al., 2021a; Sapkota et al., 2021b). In this study, RNA-sequencing was performed on the floral bud samples that were collected at five time points during endodormancy and ecodormancy, yielding 40-50 million raw reads per library, and more than 90% of the reads were successfully mapped to the apple reference genome.

**Figure 1:**
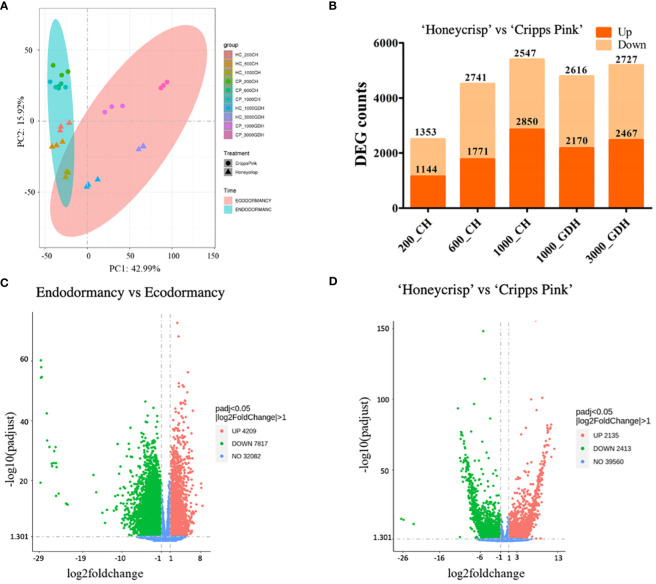
Transcriptome variance distinguishes genotypes and dormancy stages. **(A)** Principal component analysis (PCA) of samples by transcriptome profile. **(B)** Number of differentially expressed genes (DEGs) at each time points in ‘Honeycrisp’ compared to ‘Cripps Pink’. **(C, D)** Volcano plots of DEGs upregulated (red), downregulated (green), non DEGs (blue) in endodormancy compared to ecodormancy **(C)** and ‘Honeycrisp’ compared to ‘Cripps Pink’ **(D)**.

In order to comprehensively examine the transcriptomic landscape of all 30 sequenced samples, encompassing two cultivars and five time points, each with three biological replicates, we employed principal component analysis (PCA) to map the gene expression-based sample distances onto a multi-dimensional space (**Figure 1A**). The first principal component (PC1), which accounted for 42.99% of the total variance, effectively separated the samples into endodormancy and ecodormancy periods, based on the divergent patterns of CH and GDH accumulations. In contrast, the second principal component (PC2), explaining 15.92% of the variance, separated the two cultivars.

To further identify the differential gene expression profiles between samples, we used a conservative significance cutoff (p-adj < 0.05) to infer differentially expressed genes (DEGs). The DEG analysis revealed that the number of DEGs between the ‘Honeycrisp’ and ‘Cripps Pink’ cultivars was relatively low during the early stage of endodormancy (200 CH), and increased as endodormancy progressed, remaining high during ecodormancy (1000 GDH and 3000 GDH) (**Figure 1B**). The comparison between endodormancy and ecodormancy irrespective of the cultivar, revealed a much larger number of DEGs, including 4209 upregulated and 7817 downregulated genes (**Figure 1C**). Similarly, when comparing the two cultivars, irrespective of the dormancy stage, we identified a total of 2135 upregulated and 2413 downregulated DEGs (**Figure 1D**).

**Figure 2:**
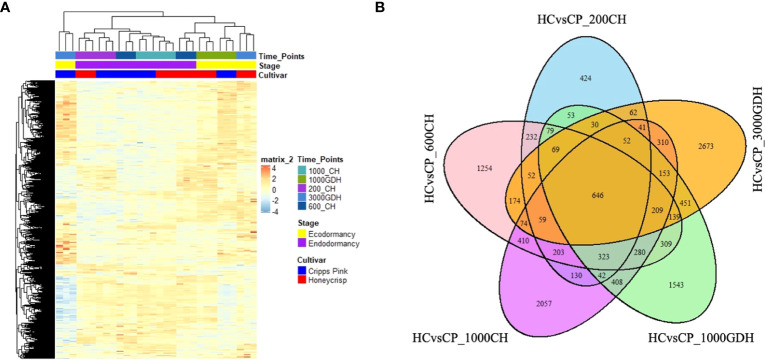
Hierarchical cluster analysis and Venn Diagrams for DEGs throughout the dormancy cycle. **(A)** Heatmap visualization of transcriptomic profiles. Rows correspond to individual transcripts, while columns represent different groups or samples. The color intensity reflects the gene expression levels (log2(FPKM+1)) among the different groups. The heatmap highlights the differentially expressed genes, identified based on their significant fold changes (|log2FoldChange| > 1) and adjusted p-values (p-adjusted < 0.05), with blue color indicating downregulation and red color representing upregulation. **(B)** a Venn Diagram showing DEGS at different time points during endo and ecodormancy in ‘Honeycrisp’ compared to ‘Cripps Pink’.

Using a hierarchical clustering method, the statistically significant DEGs were subsequently investigated to determine their expression patterns across all samples. Based on the expression levels of these DEGs, the clustering of all samples revealed five separate groups that corresponded to the different sampling time periods (**Figure 2A**). Notably, the expression profiles of endodormancy (200, 600, and 1000 CH) were significantly distinguishable from those of ecodormancy (1000 and 3000 GDH), with ecodormancy exhibiting more pronounced clustering. We plotted the DEG counts for each time point to evaluate the variation in the number of DEGs over time, as presented in **Figure 2B**. Our findings indicate high numbers of DEGs were unique to a particular time point, with only a few overlapping between or among the time points comparatively. This suggests a high degree of temporal specificity in gene expression during the dormancy transition and underscores the significance of sampling at multiple time points to capture the complete transcriptomic dynamics of this intricate process.

### Functional annotation of differentially expressed genes

We conducted a Gene Ontology (GO)-based enrichment analysis to identify the major functional categories of DEGs in our study. A threshold of adjusted p-value ≤ 0.05 was used to define significance of the GO terms. Notably, we found high number of significantly enriched GO terms at 600 CH during endodormancy and 1000 GDH and 3000 GDH during ecodormancy.

**Figure 3:**
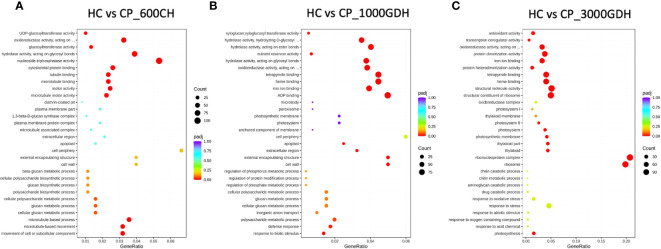
Time-series analysis of significantly enriched DEGs between ‘Honeycrisp’ and ‘Cripps Pink’ during endodormacy (**A**: 600 CH) and ecodormancy (**B** and **C**: 1000 GDH and 3000 GDH). Dot size represents the number of genes enriched in each GO at p-adj<0.05.

During endodormancy at 600 CH, the DEGs were found to be enriched for functions related to biological processes (BP) and molecular functions (MF), but not cellular component (CC) (**Figure 3A**). The top most enriched GO terms represented functions associated with mitosis, hydrolysis, and oxidation-reduction. Specifically, these terms encompassed microtubule processes, tubulin binding, skeleton protein binding, movement of cell or subcellular components, glucosyl transferase activity, hydrolase and pyrophosphatase activities, and oxidation-reduction. In contrast, during ecodormancy (1000 GDH and 3000 GDH), DEGs were enriched for functions related to all three categories: BP, MF, and CC (**Figures 3B, C**). The top GO terms identified included those related to oxidation-reduction, hydrolysis, and structural integrity, transport, and metabolism. These terms encompassed oxidoreductase activity, antioxidant activities, hydrolase activity, peroxidase activity, xyloglucan: xyloglucosyl transferase activity, proteosome complex, cell wall, heme binding, protein dimerization and structural constitution of the ribosome.

**Figure 4:**
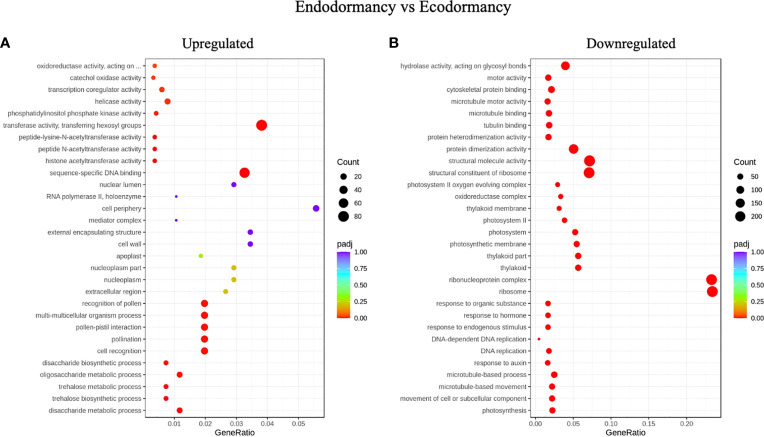
Gene ontology (GO) enrichment analysis. Top most significantly enriched GO terms of DEGs between endodormancy and ecodormancy in apple. **(A)** upregulated genes **(B)** downregulated genes in the cellular components, molecular function, and biological processes.

When we compared DEGs between endodormancy and ecodormancy, we identified an enrichment of GO terms related to transferase activity transferring hexosyl group which is related to membrane integrity, sequence specific DNA binding, oxidoreductase activity, transcriptional co-regulator that were upregulated, while terms associated with ribosome, ribonucleoprotein complex, protein dimerization, response to hormone, microtubule binding, tubulin binding, and translation were downregulated (**Figures 4A, B**).

### Co-expression network analysis

**Figure 5:**
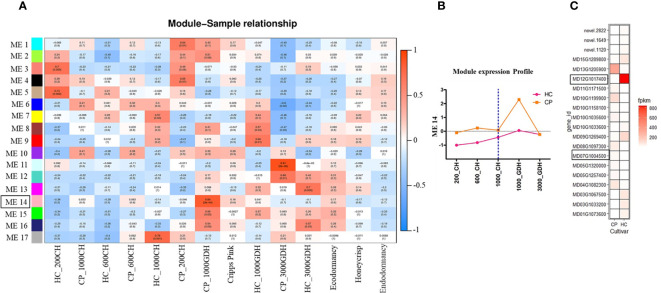
WGCNA of the floral buds of apple during endo and ecodormancy in apple cultivars ‘Honeycrisp’(HC) and ‘Cripps Pink’ (CP). **(A)** Module–sample relationships. Each row corresponds to a module eigengene, each column corresponds to a trait, and each cell consists of the corresponding correlation and p-value. **(B)** The expression profiles of ME 14 eigengenes (mean ± SE). **(C)** Expression profile of the top ten upregulated and downregulated genes at 1000 GDH in ‘HC’ and ‘CP’ based on adjusted p-value.

We employed a co-expression network analysis to gain deeper insights into the coordinated gene expression profiles across different time points, between dormancy stages, and cultivars. Using weighted gene co-expression network analysis (WGCNA), total set of DEGs were grouped into 17 distinct modules, each assigned a unique color (**Figure 5A**). A higher correlation p-value within a module indicates a higher expression level at that stage. Among these modules, module pink (ME 14) was identified as one of the most significant modules (P-value = 2e-04), positively correlated with the 1000 GDH time point. However, we did not find any module that showed contrasting differences between the two dormancy or the two cultivars.

Module pink (ME 14) was found to comprise 1162 genes, with the mean eigengene values of the genes showing a low expression initially that peaked at 1000 GDH, with a comparatively lower increase in ‘Honeycrisp’ compared to ‘Cripps Pink’ (**Figure 5B**). Further analysis of the top 20 genes within this module showed that the most contrasting genes between the two cultivars were MD12G1017400 and MD07G1004500, representing methyltransferase and lipoxygenase, respectively (**Figure 5C**).

To identify significant (p-adj <0.05) DEGs at the 1000 GDH, we merged all the genes from ME 14 and DEGs from 1000 GDH in ‘Honeycrisp’ versus ‘Cripps Pink’. We identified a total of 297 significant DEGs (**Table S2**), with the most significant DEGs downregulated in ‘Honeycrisp’ compared to ‘Cripps Pink’. These downregulated genes were mainly related to hormones, oxidation-reduction, lipid metabolism, and transmembrane transport activity (**Table 1**). Specifically, jasmonic acid-related transcription factors MYC2 and MYC4, salicylic acid-related salicylate-3 hydroxylase, FAD-dependent urate hydroxylase, linoleate 13s-lipoxygenase, peroxidase, L-ascorbate oxidase, peptide methionine transferase, ATPase 2, and vacuolar cation/proton exchanger 3 (CAX3) were among the most significant downregulated genes. These findings suggest a potential role of these genes in regulating bloom time, and future studies are needed to validate their functions.

**Table 1:** Top most significant DEGs in ME 14 comparing ‘Honeycrisp’ vs ‘Cripps Pink’.

Gene name	Gene description	log2foldchange	P-value	p adjust
MD13G1205900	Protein Sieve Element Occlusion-Related 1	-4.504603512	7.38E-110	1.39E-105
MD10G1158100	Cytochrome P450 82G1 & PF00067	-5.733170562	4.10E-45	1.93E-41
MD11G1171500	Salicylate 3-hydroxylase DLO2	-3.739393854	5.22E-44	2.19E-40
MD05G1320000	Polypeptide of LTR copia-type	-4.937456224	3.81E-42	1.44E-38
MD06G1160700	Peptide methionine sulfoxide reductase	-5.146084523	2.91E-39	6.47E-36
MD13G1206000	Protein Sieve Element Occlusion	-2.134300917	3.14E-28	2.45E-25
MD11G1182500	L-ascorbate oxidase	-2.042487085	1.54E-27	1.10E-24
MD01G1086800	Transcription factor MYC2; Jasmonate Insensitive 1	-2.344770627	4.34E-26	2.73E-23
MD14G1132200	Cellulose synthase-like protein G3	-1.911601539	1.05E-24	5.45E-22
MD10G1138400	Cytochrome P450 87A3 & PF00067	-2.561962305	4.79E-22	1.79E-19
MD11G1267000	F-box/LRR-repeat protein	-7.063748973	2.30E-19	6.49E-17
MD02G1317800	Linoleate 13S-lipoxygenase 2-1	-2.085688719	5.39E-19	1.46E-16
MD09G1102600	Xyloglucan endotransglucosylase/hydrolase protein 9	-1.45855265	2.03E-18	4.88E-16
MD05G1123600	FAD-dependent urate hydroxylase	-4.817490102	3.23E-18	7.53E-16
MD00G1112500	Peroxidase 51	-4.184489271	7.13E-18	1.60E-15
MD09G1089200	(R,S)-reticuline 7-O-methyltransferase	-2.580208424	3.90E-17	7.62E-15
MD17G1164400	Jasmonate ZIM domain-containing protein 2	-5.942708159	1.79E-16	3.13E-14
MD01G1086900	Transcription factor MYC2	-4.040837816	7.40E-16	1.19E-13
MD08G1085600	Calcium-transporting ATPase 2	-4.359118072	1.36E-14	1.83E-12
MD03G1070900	Vacuolar cation/proton exchanger 3	-2.137587737	7.46E-12	5.94E-10
MD14G1137200	Transcription factor MYC4	-2.266555879	1.92E-10	1.14E-08

### Gene expression analyses by qRT-PCR

**Figure 6:**
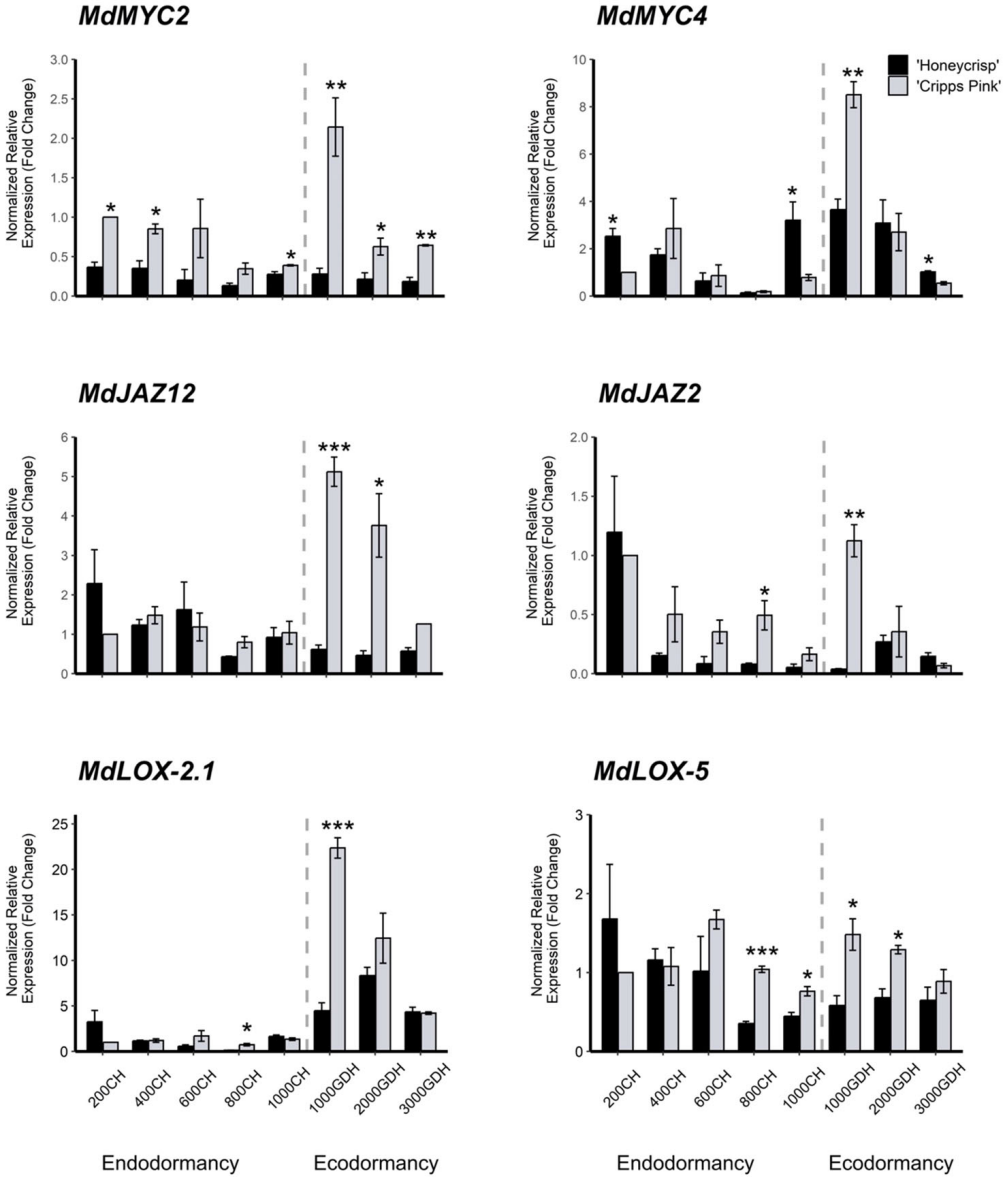
Expression profile of MdMYC, MdJAZ, and MdLOX genes at endodormancy and ecodormancy in ‘Honeycrisp’ and ‘Cripps Pink’. The expression of each gene was normalized to that of two reference genes (MdActin and *MdGAPDH*) and then calculated relative to the expression level in the control sample (‘Cripps Pink’ at 200 CH). Error bars represent the standard error of the means of three biological and three technical replicates. CH and GDH refers to Chilling Hour and Growing Degree Hour, respectively. *,**,*** represent significant at P-value = 0.05, 0.01, 0.001, respectively.

In this study, we examined the differential expression of six genes associated with the jasmonic acid biosynthesis and signaling pathway. The genes, *MdMYC2* (MD01G1086900), *MdMYC4* (MD14G1137200), *MdJAZ12* (MD15G1434400), *MdJAZ2* (MD17G1164400), MdLOX-2.1 (MD02G1317800), and *MdLOX-5* (MD04G1204200), were selected from the significant DEG list in the ME 14 module. Generally, these genes exhibited a similar expression pattern, with lower levels during endodormancy and increased expression at the onset of ecodormancy (around 1000 GDH), except for *MdLOX-5*, which showed slightly elevated expression at 200 CH and 600 CH. Moreover, we found that the expression levels of all genes were significantly higher in ‘Cripps Pink’ than in ‘Honeycrisp’ at all dormancy stages. Specifically, *MdMYC2* showed significantly higher expression levels in ‘Cripps Pink’ at 200 CH, 400 CH, 1000 CH, 1000 GDH, 2000 GDH, and 3000 GDH, while *MdMYC4* was only elevated in ‘Cripps Pink’ at 1000 GDH, but higher in ‘Honeycrisp’ at 200 CH and 1000 CH. For the JAZ family genes, *MdJAZ12* was significantly highly expressed in ‘Cripps Pink’ at 1000 GDH and 2000 GDH, whereas MdJAZ2 was significantly higher in ‘Cripps Pink’ at 800 CH and 1000 GDH. *MdLOX-2.1* and *MdLOX-5* showed similar expression patterns, with significantly higher expression of *MdLOX-2.1* in ‘Cripps Pink’ at 800 CH and 1000 GDH and significantly elevated expression of *MdLOX-5* in ‘Cripps Pink’ at 800 CH, 1000 CH, 1000 GDH, and 2000 GDH (**Figure 6**). In order to validate the RNA-Seq results for the six studied genes, the expression values obtained form qRT-PCR were correlated to FPKM values from RNA-Seq. The correlation coefficient alongside the p-values is presented in **Table S3**. All genes except *MdMYC2* and *MdJAZ12* showed significant correlation at p-value ≤ 0.05 (**Table S3**), indicating that these genes exhibited similar expression pattern in both datasets.

## Discussion

Global climate change has disrupted spring phenology in recent years, resulting in an increased risk of frost damage in early blooming apple cultivars. Bud dormancy is a crucial stage in the life cycle of temperate perennials as it directly affects the bud re-growth in spring and flowering time. To identify candidate genes regulating flowering time, this study analyzed transcript expression profiles during the dormancy-regrowth cycle in two apple cultivars; ‘Cripps Pink’ and ‘Honeycrisp’; that represent early- and late-bloom cultivars, respectively. Our findings suggest that despite the number of active pathways during the dormancy-regrowth cycle, the fundamental factors controlling the release of dormancy is a complicated interplay between hormones, lipid metabolism, redox balance, and calcium signaling, mainly during ecodormancy.

To compare differentially expressed genes (DEGs) between the two apple cultivars, a co-expression network analysis was conducted. However, no modules were identified that could distinguish between the cultivars irrespective of dormancy stages, or between dormancy stages irrespective of cultivars. Nonetheless, a time series transcriptomic analysis revealed significant modules between the cultivars, particularly during ecodormancy. This could be due to the fact that the two cultivars had similar endodormancy release times and differs only in their ecodormancy release time (Sapkota et al., 2021a). The most significant module, ME 14, was positively correlated with ecodormancy in ‘Cripps Pink’ but negatively correlated with ‘Honeycrisp’. This suggests that the genes within ME 14 may play a crucial role in regulating bloom time. The most significant DEGs from this module were mainly related to hormonal signaling, oxidation-reduction, fatty acid degradation, and calcium signaling.

The ME 14 has been subjected to further analysis, revealing 405 differentially expressed genes (DEGs) between the two cultivars at 1000 GDH. Notably, among the top 20 most significant DEGs were genes related to JA biosynthesis (e.g., *MdLOX-2.1*) and signaling (e.g., *MdMYC2*, *MdMYC4*, and *MdJAZ2*), with additional JA-related genes (*MdLOX-5* and *MdJAZ12*) identified upon examination of the full list of DEGs at that time point. Strikingly, all JA-related genes exhibited significantly reduced expression in the ‘Honeycrisp’ cultivar. Validation by qRT-PCR confirmed the RNA-Seq data, indicating significantly higher levels of JA-related genes in the early-bloom cultivar during the ecodormancy stage, particularly at 1000 GDH. Moreover, the analysis identified SA 3-hydroxylase (MD11G1171500), a gene involved in SA catabolism, as one of the 20 most significant DEGs at 1000 GDH, with significantly higher expression in the early-bloom cultivar. Given the antagonistic relationship between JA and SA pathways, as evidenced in several previous studies (reviewed by Li et al., 2019), upregulation of SA 3-hydroxylase could potentially induce JA biosynthesis. Taken together, these findings suggest that the JA pathway plays a crucial role in flower time regulation in apple, consistent with similar studies in beech trees (Juvany et al., 2015), sweet cherry (Ionescu et al., 2017) and peach (Liu et al., 2021) where higher JA levels associated with bud burst. Furthermore, JA has been shown to affect several aspects of flower development including flower opening (Sherif et al., 2015; Huang et al., 2023), stamen development (Feys et al., 1994; Ishiguro et al., 2001), anther dehiscence (Xiao et al., 2014), male sex determination (Acosta et al., 2009), and maternal control of seed maturation (Li et al., 2004), further underscoring its significance in the context of phenotypic differences between ‘Cripps Pink’ and ‘Honeycrisp’ during the ecodormancy period.

The alteration of the cell membrane’s fatty acid and lipid composition is a crucial process during bud dormancy. This change is essential for protecting cells against dehydration caused by freezing (Wang and Faust, 1990; Uemura and Steponkus, 1999). Moreover, the fatty acid composition within a bud can indicate its dormancy stage. For instance, linoleic acid (C18:2) accumulation is often linked to dormancy maintenance, while linolenic acid (C18:3) accumulation is associated with dormancy release in peach buds (Erez et al., 1997). Indeed, applying α-linolenic acid encourages dormancy break in Japanese pear (Sakamoto et al., 2010). This acid is also a precursor for jasmonic acid (JA), which plays a role in dormancy release and flowering. Another oxylipin metabolite, derived from α-linolenic acid via a 9-specific lipoxygenase, is 9-hydroxy-10-oxo-12(Z), 15(Z)-octadecadienoic acid (KODA). This compound has been found to induce bud break in Japanese pear when applied (Sakamoto et al., 2010). Furthermore, inoculating peach plants with the peach latent viroid has been shown to inhibit fatty acid unsaturation, particularly the conversion of linoleic acid (C18:2) to α-linolenic acid (C18:3), resulting in delayed dormancy release (Gibson et al., 2004).These findings collectively suggest that linolenic acid accumulation and its oxylipin metabolites, including JA, may play a role in regulating dormancy release and flowering. In the current study, we discovered that the linoleate 13S-lipoxygenase transcripts (*MdLOX-2.1* and *MdLOX-5*) were downregulated in ‘Honeycrisp’ compared to ‘Cripps Pink’ during ecodormancy. This finding implies reduced levels of α-linolenic acid in ‘Honeycrisp,’ which could contribute to its late-bloom phenotype.

In addition to changes in JA biosynthesis, the increase in unsaturation of fatty acids to α-linolenic acid may also result in membrane depolarization, leading to calcium uptake through the activation of calcium channels (Miedema et al., 2001; Penzo et al., 2002). Considine and Foyer (2021) proposed that this calcium channel activation could initiate a growth signal via mitogen-activated protein kinase (MAPK) pathways. These MAPK pathways have been linked to a range of cellular reactions, such as cell division, differentiation, and hormonal responses (Sinha et al., 2011). In fact, our research pinpointed differentially expressed receptor protein kinases, like serine/threonine protein kinase (MD10G1201080, MD16G101560) and cystine-rich protein kinase (MD13G1096600). (**Table 1**), which might function upstream of MAP kinase and were also found to be downregulated in the late-flowering ‘Honeycrisp’ cultivar.

The process of converting linoleic acid (C18:2) to linolenic acid (C18:3) by fatty acid desaturase necessitates a heightened level of reducing power (specifically, NADPH) and active oxygen (O_2_). Reactive oxygen species (ROS), particularly H_2_O_2_, are generated as a byproduct of this unsaturation reaction. The accumulation of H_2_O_2_ during bud dormancy has been widely reported in numerous species and is considered a vital signaling molecule for dormancy release (Beauvieux et al., 2018). Exogenous H_2_O_2_ has been shown to act as a substitute for chilling (Pérez and Burgos, 2004; Kuroda et al., 2005; Pérez et al., 2008). Conversely, inhibiting NADPH oxidase activity in potato tubers has been found to lead to decreased ROS production and delayed dormancy release (Liu et al., 2017). Our previous studies on peach and apple have also revealed the accumulation of H_2_O_2_ during endodormancy, with the peak occurring at the time of endodormancy release, followed by a decline during ecodormancy (Islam et al., 2021; Sapkota et al., 2021b). Interestingly, our investigations have indicated that the levels of H_2_O_2_ and NADPH oxidase activity peaked earlier (at 1000 CH) and more intensely in ‘Cripps Pink’ than in ‘Honeycrisp’ (Sapkota et al., 2021a). To counteract the elevated H_2_O_2_ levels in ‘Cripps Pink’, we recorded a significant increase in catalase and glutathione reductase enzyme activity at 1000 CH (Sapkota et al., 2021b). Additionally, peroxidase and ascorbate can aid in ROS detoxification. In this study, we observed a significant downregulation of peroxidase transcripts (Md00G112500) and ascorbate oxidase transcripts (Md11G1182500) in ‘Honeycrisp’ compared to ‘Cripps Pink’. These results support our previous findings on the delayed and lower H_2_O_2_ levels in ‘Honeycrisp’ (Sapkota et al., 2021b).

The plasma membrane-based NADPH oxidase harbors a Ca^2+^ binding motif in its N-terminal. The binding of Ca^2+^ activates the NADPH oxidase, and through positive feedback regulation, it triggers the activation of calcium channels (Takeda et al., 2008). Functional analysis of the genes *rbohD* and *rbohF*, which are associated with NADPH oxidase in Arabidopsis, revealed that NADPH oxidase is essential not only for ROS production but also for JA-signaling through MYC2 (Maruta et al., 2011). In the present study, along with the downregulation of *MYC2* transcript in ‘Honeycrisp’, the transcripts of vacuolar cation/proton exchanger 3 (Md03G1070900) and Ca^2+^-ATPase 2 (Md08G1085600) were also downregulated compared to the early-blooming ‘Cripps Pink’. It is worth noting that in grapes, the induction of bud break by hydrogen cyanamide is linked to transient oxidative stress that activates the transcription of the calcium-transporting Ca^2+^-ATPase gene (Or et al., 2000), while in sweet cherry, it is associated with increased JA levels (Ionescu et al., 2017). Our earlier findings, as reported in Sapkota et al., 2021a; Sapkota et al., 2021b, also align with the current observation that a relatively higher level of H_2_O_2_ at 1000 CH corresponds to an increased level of JA-Ile in ‘Cripps Pink’ when compared to ‘Honeycrisp’. Nonetheless, we noticed a more rapid decline in H_2_O_2_ in the early blooming cultivar, which corresponded to a higher level of catalase (Sapkota et al., 2021b). Overall, this points towards a complex interplay among fatty acid metabolism, redox balance, calcium channel, and JA acid biosynthesis and signaling in the regulation of bud burst and flowering time in apple.

## Conclusion

This study has provided valuable insights into the molecular regulation of bud dormancy and flowering time in apple cultivars, which is crucial for understanding the impact of global climate change on spring phenology and frost damage risk. Our findings reveal that the release of dormancy and regulation of flowering time in apple is a complex interplay of hormonal signaling, lipid metabolism, redox balance, and calcium signaling, predominantly during ecodormancy. The transcriptomic analysis has highlighted the potential role of JA pathway and its association with α-linolenic acid metabolism in regulating bloom time. Moreover, our findings have identified additional genes and pathways, such as fatty acid degradation, oxidation-reduction, and calcium signaling, which could contribute to the phenotypic differences between the early-blooming ‘Cripps Pink’ and late-blooming ‘Honeycrisp’ apple cultivars. This research offers a foundation for future studies to further unravel the complexity of molecular mechanisms controlling bud dormancy and flowering time in apple and other temperate perennial species. This understanding can ultimately lead to the development of more resilient cultivars with improved adaptation to changing climate conditions and reduced risk of frost damage.

## Data availability statement

The datasets presented in this study can be found in online repositories. The names of the repository/repositories and accession number(s) can be found in the article/**Supplementary Material**. The authors will provide the codes/scripts upon request.

## Author contributions

SS investigation, methodology, formal analysis, validation, writing–original draft preparation. MS and KRJ methodology, software. TSA resources, supervision. SMS conceptualization, project administration, funding acquisition, supervision, data curation, writing–review and editing. All authors contributed to the article and approved the submitted version.
