# Supplementary material for: From endodormancy to ecodormancy: the transcriptional landscape of apple floral buds
Source: Front Plant Sci. 2023 Jul 14;14:1194244. doi: 10.3389/fpls.2023.1194244 (PMC10375413; doi:10.3389/fpls.2023.1194244)
Supplement: Supplementary file 1 [file DataSheet_1.pdf]

Supplementary Material

From Endodormancy to Ecodormancy: The Transcriptional Landscape of Apple Floral Buds

Sangeeta Sapkota[1, 2], Mohamed Salem[3], Khalil Jahed[1], Timothy S. Artlip[4], Sherif M. Sherif[1*]

[1]Alson H. Smith Jr. Agriculture Research and Extension Center, School of Plant and Environmental Sciences, Virginia Tech, Winchester, VA, United States

[2]Department of Horticulture, Michigan State University, East Lansing, MI, United States

[3]Department of Statistics, Virginia Tech, Blacksburg, VA, United States

[4]Appalachian Fruit Research Station, United States Department of Agriculture—Agriculture Research Service, Kearneysville, WV, United States

[*] ***Corresponding author:***

Sherif M. Sherif; *E-mail address: ssherif@vt.edu; Tel: +1 (540)- 232-6035*

Table S1: List of primers used for RT-qPCR. Gene description, their accession code, forward and reverse sequence are presented

		Primer Sequence	
Gene Description/ Acronym	Accession code	Forward	Reverse
MdMYC2	MD01G1086900	GACGTCGGTGGATCAAACG	TGGCGTCCGAACACACTAT
MdMYC4	MD14G1137200	ACGCACTCAAAGACCTGGA	CCATGGGGCACTCTAACCA
MdJAZ12	MD15G1434400	ACCACCTCTTAGACCGCAA	GCCACGACGCTTTTCAAGA
MdJAZ2	MD17G1164400	GCAACCTGCTGAGCCAATT	AATCAGCAGCCTCCATGGT
MdLOX-2.1	MD02G1317800	AGTGAAGGGGTATGGGCAC	ACCTCTCCAAACCCTGCTG
MdLOX-5	MD04G1204200	CAATCCAGATCGTGCCACG	TCATGCACAAGAAACCGGC
MdGAPDH	MD13G1111500	ACGTGCTGCTTCCTTCAAC	TCCGGTCAACTTTCCGTTA
MdActin	MD04G1127400	CCGTGTTCCCTAGCATTGTT	CAGGAGCAACACGAAGTTCA

Table S2. List of the differentially expressed genes (DEGs) in ME_14 between 'Honeycrisp' and 'Cripps Pink'

GeneID	log2foldchange	padjust	gene_description	gene_chr	gene_start	gene_end	gene_strand	gene_length
MD13G1205900.v1.1	-4.504604	1.39E-105	- && Q9SS87.1 RecName: Full=Protein SIEVE ELEMENT OCCLUSION B; Short=AtSEOb; AltName: Full=Protein SIEVE ELEMENT OCCLUSION-RELATED 1; Short=AtSEOR1 && PF14577:Sieve element occlusion C-terminus	Chr13	18659915	18661199	+	753
MD10G1158100.v1.1	-5.733171	1.93E-41	- && Q9LSF8.1 RecName: Full=Cytochrome P450 82G1 && PF00067:Cytochrome P450	Chr10	24694444	24696651	-	1623
MD11G1171500.v1.1	-3.739394	2.19E-40	- && Q9ZSA7.1 RecName: Full=Protein DMR6-LIKE OXYGENASE 2; AltName: Full=2-oxoglutarate (2OG)-Fe(II) oxygenase-like protein DLO2; AltName: Full=Salicylate 3-hydroxylase DLO2; Short=S3H DLO2; Short=SA 3-hydroxylase DLO2; Short=Salicylic acid 3-hydroxylase DLO2 && PF14226:non-haem dioxygenase in morphine synthesis N-terminal|PF03171:2OG-Fe(II) oxygenase superfamily	Chr11	18680045	18681761	+	1062
MD05G1320000.v1.1	-4.937456	1.44E-38	- && - && PF14223:gag-polypeptide of LTR copia-type	Chr05	44742445	44743921	+	885
MD06G1160700.v1.1	-5.146085	6.47E-36	- && P54152.2 RecName: Full=Peptide methionine sulfoxide reductase; AltName: Full=Fruit-ripening protein E4; AltName: Full=Peptide-methionine (S)-S-oxide reductase; Short=Peptide Met(O) reductase; AltName: Full=Protein-methionine-S-oxide reductase && PF01625:Peptide methionine sulfoxide reductase	Chr06	30196820	30206164	+	672
MD04G1011700.v1.1	-3.815522	3.63E-27	- && Q40392.1 RecName: Full=TMV resistance protein N && PF00931:NB-ARC domain|PF01582:TIR domain	Chr04	1324708	1331182	-	3183
MD13G1206000.v1.1	-2.134301	2.45E-25	- && Q9SS87.1 RecName: Full=Protein SIEVE ELEMENT OCCLUSION B; Short=AtSEOb; AltName: Full=Protein SIEVE ELEMENT OCCLUSION-RELATED 1; Short=AtSEOR1 && PF14576:Sieve element occlusion N-terminus|PF14577:Sieve element occlusion C-terminus	Chr13	18719748	18723375	+	2049
MD11G1182500.v1.1	-2.042487	1.10E-24	- && Q40588.1 RecName: Full=L-ascorbate oxidase; Short=ASO; Short=Ascorbase; Flags: Precursor && PF07731:Multicopper oxidase|PF00394:Multicopper oxidase|PF07732:Multicopper oxidase	Chr11	22479062	22485544	+	1764
MD01G1086800.v1.1	-2.344771	2.73E-23	- && Q39204.2 RecName: Full=Transcription factor MYC2; Short=AtMYC2; AltName: Full=Basic helix-loop-helix protein 6; Short=AtbHLH6; Short=bHLH 6; AltName: Full=Protein JASMONATE INSENSITIVE 1; AltName: Full=R-homologous Arabidopsis protein 1; Short=RAP-1; AltName: Full=Transcription factor EN 38; AltName: Full=Z-box binding factor 1 protein; AltName: Full=bHLH transcription factor bHLH006; AltName: Full=rd22BP1 && PF00010:Helix-loop-helix DNA-binding domain|PF14215:bHLH-MYC and R2R3-MYB transcription factors N-terminal	Chr01	19581044	19582534	+	1491
MD14G1132200.v1.1	-1.911602	5.45E-22	- && Q0WVN5.2 RecName: Full=Cellulose synthase-like protein G3; Short=AtCslG3 && PF03552:Cellulose synthase	Chr14	21023525	21028150	+	2208
MD10G1138400.v1.1	-2.561962	1.79E-19	- && Q7XU38.3 RecName: Full=Cytochrome P450 87A3 && PF00067:Cytochrome P450	Chr10	22312875	22322581	-	1434
MD11G1267000.v1.1	-7.063749	6.49E-17	- && Q9LX51.1 RecName: Full=F-box/LRR-repeat protein At3g59200 && PF00646:F-box domain|PF08387:FBD	Chr11	38206055	38210003	+	1503

MD02G1317800.v1.1	-2.085689	1.46E-16	- && O24370.1 RecName: Full=Linoleate 13S-lipoxygenase 2-1, chloroplastic; AltName: Full=Lipoxygenase 2-1; Flags: Precursor && PF00305:Lipoxygenase|PF01477:PLAT/LH2 domain	Chr02	37219640	37227516	+	2745
MD09G1102600.v1.1	-1.458553	4.88E-16	- && Q8LDW9.2 RecName: Full=Xyloglucan endotransglucosylase/hydrolase protein 9; Short=At-XTH9; Short=XTH-9; Flags: Precursor && PF06955:Xyloglucan endo-transglycosylase (XET) C-terminus|PF00722:Glycosyl hydrolases family 16	Chr09	7492197	7495383	-	891
MD05G1123600.v1.1	-4.81749	7.53E-16	- && Q6F6Y2.1 RecName: Full=FAD-dependent urate hydroxylase; AltName: Full=Flavoprotein urate hydroxylase && PF01494:FAD binding domain	Chr05	24598388	24601129	-	1248
MD00G1112500.v1.1	-4.184489	1.60E-15	- && Q9SZE7.1 RecName: Full=Peroxidase 51; Short=Atperox P51; AltName: Full=ATP37; Flags: Precursor && PF00141:Peroxidase	Chr00	23677049	23679547	-	978
novel.3247	-3.687724	6.37E-15	-	Chr12	19095947	19096766	-	820
MD09G1089200.v1.1	-2.580208	7.62E-15	- && Q6WUC2.1 RecName: Full=(R,S)-reticuline 7-O-methyltransferase; Short=7OMT && PF08100:Dimerisation domain|PF00891:O-methyltransferase	Chr09	6502568	6503997	-	1095
MD17G1164400.v1.1	-5.942708	3.13E-14	- && Q9S7M2.1 RecName: Full=Protein TIFY 10B; AltName: Full=Jasmonate ZIM domain-containing protein 2 && PF09425:Divergent CCT motif|PF06200:tify domain	Chr17	16289064	16290315	-	642
MD01G1086900.v1.1	-4.040838	1.19E-13	- && Q39204.2 RecName: Full=Transcription factor MYC2; Short=AtMYC2; AltName: Full=Basic helix-loop-helix protein 6; Short=AtbHLH6; Short=bHLH 6; AltName: Full=Protein JASMONATE INSENSITIVE 1; AltName: Full=R-homologous Arabidopsis protein 1; Short=RAP-1; AltName: Full=Transcription factor EN 38; AltName: Full=Z-box binding factor 1 protein; AltName: Full=bHLH transcription factor bHLH006; AltName: Full=rd22BP1 && PF14215:bHLH-MYC and R2R3-MYB transcription factors N-terminal|PF00010:Helix-loop-helix DNA-binding domain	Chr01	19597538	19599016	+	1479
MD15G1248200.v1.1	-1.963193	3.78E-13	- && - && PF12681:Glyoxalase-like domain	Chr15	20692664	20693835	+	555
MD08G1085600.v1.1	-4.359118	1.83E-12	- && O81108.1 RecName: Full=Calcium-transporting ATPase 2, plasma membrane-type; AltName: Full=Ca(2+)-ATPase isoform 2 && PF00689:Cation transporting ATPase, C-terminus|PF12515:Ca2+-ATPase N terminal autoinhibitory domain|PF12710:haloacid dehalogenase-like hydrolase|PF00122:E1-E2 ATPase|PF00690:Cation transporter/ATPase, N-terminus	Chr08	7104881	7119775	+	3501
MD00G1140000.v1.1	-2.155985	1.97E-12	- && Q39134.2 RecName: Full=Amino acid permease 3; AltName: Full=Amino acid transporter AAP3 && PF01490:Transmembrane amino acid transporter protein	Chr00	30551515	30555417	+	1470
novel.1036	-4.155718	4.95E-12	-	Chr04	2425531	2429810	-	1628
MD05G1319800.v1.1	-2.471227	7.53E-12	- && P43309.1 RecName: Full=Polyphenol oxidase, chloroplastic; Short=PPO; AltName: Full=Catechol oxidase; Flags: Precursor && PF12142:Polyphenol oxidase middle domain|PF12143:Protein of unknown function (DUF_B2219)|PF00264:Common central domain of tyrosinase	Chr05	44733185	44734948	+	1764
MD15G1236600.v1.1	-2.737797	8.40E-12	- && Q38853.1 RecName: Full=Rhodanese-like domain-containing protein 15, chloroplastic; AltName: Full=Protein DARK INDUCIBLE 1; AltName: Full=Senescence-associated protein 1; Short=AtSEN1; AltName: Full=Sulfurtransferase 15; Short=AtStr15; Flags: Precursor && PF00581:Rhodanese-like domain	Chr15	19456646	19459912	+	546

MD15G1264200.v1.1	-2.082998	5.48E-11	- && - && PF01925:Sulfite exporter TauE/SafE	Chr15	22646734	22650279	-	1455
MD14G1024700.v1.1	-2.151158	8.44E-11	- && Q9LHA8.1 RecName: Full=Probable mediator of RNA polymerase II transcription subunit 37c; AltName: Full=Heat shock 70 kDa protein 4; AltName: Full=Heat shock cognate 70 kDa protein 4; AltName: Full=Heat shock cognate protein 70-4; Short=AtHsc70-4; AltName: Full=Heat shock protein 70-4; Short=AtHsp70-4 && PF00012:Hsp70 protein	Chr14	2295124	2296382	-	948
MD03G1070900.v1.1	-2.137588	5.94E-10	- && Q93Z81.1 RecName: Full=Vacuolar cation/proton exchanger 3; AltName: Full=Ca(2+)/H(+) antiporter CAX3; AltName: Full=Ca(2+)/H(+) exchanger 3; AltName: Full=Protein CATION EXCHANGER 3 && PF01699:Sodium/calcium exchanger protein	Chr03	5786105	5790084	+	1356
MD03G1209600.v1.1	-3.478961	1.33E-09	- && Q9SK27.2 RecName: Full=Early nodulin-like protein 1; AltName: Full=Phytocyanin-like protein; Flags: Precursor && PF02298:Plastocyanin-like domain	Chr03	28890946	28892432	-	618
MD15G1343500.v1.1	-2.028737	1.33E-09	- && Q39134.2 RecName: Full=Amino acid permease 3; AltName: Full=Amino acid transporter AAP3 && PF01490:Transmembrane amino acid transporter protein	Chr15	39935484	39938860	-	1470
MD12G1025600.v1.1	-2.575231	1.83E-09	- && P09189.1 RecName: Full=Heat shock cognate 70 kDa protein && PF10712:NAD-specific glutamate dehydrogenase|PF00012:Hsp70 protein	Chr12	2737744	2741109	-	1548
MD14G1019600.v1.1	-1.710185	2.46E-09	- && Q9LZS7.1 RecName: Full=GDSL esterase/lipase At5g03610; AltName: Full=Extracellular lipase At5g03610; Flags: Precursor && PF00657:GDSL-like Lipase/Acylhydrolase	Chr14	1856112	1859691	-	1074
MD08G1051100.v1.1	-4.366834	7.40E-09	- && Q8S9J6.1 RecName: Full=Aspartyl protease family protein At5g10770; Flags: Precursor && PF14541:Xylanase inhibitor C-terminal|PF14543:Xylanase inhibitor N-terminal	Chr08	4037411	4039621	-	1494
MD14G1137200.v1.1	-2.266556	1.14E-08	- && O49687.1 RecName: Full=Transcription factor MYC4; Short=AtMYC4; AltName: Full=Basic helix-loop-helix protein 4; Short=AtbHLH4; Short=bHLH 4; AltName: Full=Transcription factor EN 37; AltName: Full=bHLH transcription factor bHLH004 && PF00010:Helix-loop-helix DNA-binding domain|PF14215:bHLH-MYC and R2R3-MYB transcription factors N-terminal	Chr14	22327588	22329531	-	1446
MD12G1039300.v1.1	-2.672021	1.45E-08	- && Q8GT20.1 RecName: Full=Benzyl alcohol O-benzoyltransferase; AltName: Full=Benzoyl coenzyme A:benzyl alcohol benzoyl transferase && PF02458:Transferase family	Chr12	4259222	4259374	-	153
MD11G1074900.v1.1	-1.942767	1.45E-08	- && Q93Z81.1 RecName: Full=Vacuolar cation/proton exchanger 3; AltName: Full=Ca(2+)/H(+) antiporter CAX3; AltName: Full=Ca(2+)/H(+) exchanger 3; AltName: Full=Protein CATION EXCHANGER 3 && PF01699:Sodium/calcium exchanger protein	Chr11	6348409	6352616	+	1362
MD09G1038100.v1.1	-1.333553	1.60E-08	- && Q9LFR3.1 RecName: Full=Gibberellin-regulated protein 14; AltName: Full=GAST1 protein homolog 14; Flags: Precursor && PF02704:Gibberellin regulated protein	Chr09	2324761	2326415	+	789
MD00G1152400.v1.1	3.2438644	1.94E-08	- && Q8L5A7.1 RecName: Full=Cytosolic sulfotransferase 15; Short=AtSOT15; AltName: Full=Sulfotransferase 2a; Short=AtST2a && PF00685:Sulfotransferase domain	Chr00	33407618	33408682	-	1065
MD02G1183900.v1.1	-1.608639	2.69E-08	- && Q8VYE5.1 RecName: Full=Glucan endo-1,3-beta-glucosidase 12; AltName: Full=(1->3)-beta-glucan endohydrolase 12; Short=(1->3)-beta-glucanase 12; AltName: Full=Beta-1,3-endoglucanase 12; Short=Beta-1,3-glucanase 12; Flags: Precursor && PF07983:X8 domain	Chr02	16681557	16683335	-	1227
novel.2656	-4.090992	3.75E-08	-	Chr10	17316903	17317503	-	601

MD09G1110500.v1.1	-1.611805	3.93E-08	- && Q00624.1 RecName: Full=L-ascorbate oxidase homolog; Flags: Precursor && PF07731:Multicopper oxidase|PF00394:Multicopper oxidase|PF07732:Multicopper oxidase	Chr09	8214979	8218113	+	1629
MD00G1085000.v1.1	-3.577173	4.62E-08	- && - && PF03248:Rer1 family	Chr00	17074339	17076472	-	261
MD09G1011400.v1.1	-2.452078	5.71E-08	- && Q9FMV7.1 RecName: Full=Cytochrome P450 94B1 && PF00067:Cytochrome P450	Chr09	753957	755501	+	1545
MD04G1176300.v1.1	-2.97079	6.54E-08	- && Q9LZV3.1 RecName: Full=Mannan endo-1,4-beta-mannosidase 6; AltName: Full=Beta-mannanase 6; AltName: Full=Endo-beta-1,4-mannanase 6; Short=AtMAN6; Flags: Precursor && PF00150:Cellulase (glycosyl hydrolase family 5)	Chr04	26710107	26712219	-	1428
MD07G1051800.v1.1	-1.776568	7.42E-08	- && Q9FPT1.2 RecName: Full=Ubiquitin carboxyl-terminal hydrolase 12; AltName: Full=Deubiquitinating enzyme 12; Short=AtUBP12; AltName: Full=Ubiquitin thioesterase 12; AltName: Full=Ubiquitin-specific-processing protease 12 && PF00917:MATH domain	Chr07	4753808	4758312	+	1479
MD14G1207400.v1.1	-2.659772	8.74E-08	- && Q9LK99.1 RecName: Full=Ras-related protein RABA1g; Short=AtRABA1g && PF00071:Ras family	Chr14	29440073	29441111	-	450
MD10G1305000.v1.1	-2.972948	1.01E-07	- && Q9M0Y3.1 RecName: Full=Equilibrative nucleotide transporter 3; Short=AtENT3; AltName: Full=Nucleoside transporter ENT3; AltName: Full=Protein FLUOROURIDINE RESISTANT 1 && PF01733:Nucleoside transporter	Chr10	39126011	39129069	-	1275
MD09G1287500.v1.1	-1.824883	1.27E-07	- && - && -	Chr09	36750571	36752491	-	1089
MD01G1194700.v1.1	-1.081636	1.64E-07	- && Q9FJ95.1 RecName: Full=Sorbitol dehydrogenase; AltName: Full=L-iditol 2-dehydrogenase && PF00107:Zinc-binding dehydrogenase|PF08240:Alcohol dehydrogenase GroES-like domain	Chr01	29342987	29344698	+	1107
MD03G1006600.v1.1	-1.7163	1.91E-07	- && Q9LHN7.1 RecName: Full=Probable polyamine transporter At3g13620 && PF13520:Amino acid permease	Chr03	541745	543668	-	1380
novel.3408	-2.054845	2.60E-07	-	Chr13	34353860	34355635	+	1776
MD09G1231400.v1.1	-1.794289	3.67E-07	- && Q5RFV4.1 RecName: Full=Probable E3 ubiquitin-protein ligase RNF144A-A; AltName: Full=RING finger protein 144A-A && PF01485:IBR domain, a half RING-finger domain	Chr09	28644245	28647751	-	951
MD04G1204200.v1.1	-1.51763	3.84E-07	- && Q43191.1 RecName: Full=Probable linoleate 9S-lipoxygenase 5; AltName: Full=Leaf lipoxygenase && PF00305:Lipoxygenase|PF01477:PLAT/LH2 domain	Chr04	29027621	29031768	-	2802
MD02G1110500.v1.1	-1.671301	4.78E-07	- && - && PF12681:Glyoxalase-like domain	Chr02	8988478	8989766	+	555
MD07G1083500.v1.1	-1.59528	6.42E-07	- && Q8LPS2.1 RecName: Full=Protein ACCELERATED CELL DEATH 6 && PF13962:Domain of unknown function|PF12796:Ankyrin repeats (3 copies)	Chr07	8411835	8414818	+	2058
novel.12	-3.636591	1.10E-06	-	Chr00	1791904	1792998	+	571
MD00G1099600.v1.1	-2.307822	1.14E-06	- && O48850.2 RecName: Full=Vesicle-associated membrane protein 725; Short=AtVAMP725 && PF00957:Synaptobrevin|PF13774:Regulated-SNARE-like domain	Chr00	21028560	21032563	+	663
MD06G1161800.v1.1	-1.778278	1.44E-06	- && Q9SS87.1 RecName: Full=Protein SIEVE ELEMENT OCCLUSION B; Short=AtSEOb; AltName: Full=Protein SIEVE ELEMENT OCCLUSION-RELATED 1; Short=AtSEOR1 && PF14576:Sieve element occlusion N-terminus|PF14577:Sieve element occlusion C-terminus	Chr06	30268058	30271310	-	2139
MD09G1088100.v1.1	-1.873587	2.01E-06	- && - && PF14244:gag-polypeptide of LTR copia-type|PF13962:Domain of unknown function	Chr09	6341710	6348154	-	2262

MD01G1137700.v1.1	-1.519125	2.36E-06	- && Q9M2S4.1 RecName: Full=L-type lectin-domain containing receptor kinase S.4; Short=LecRK-S.4; Flags: Precursor && PF00139:Legume lectin domain|PF00069:Protein kinase domain	Chr01	24741386	24743467	-	2082
MD12G1012700.v1.1	-1.596326	3.33E-06	- && Q9FDW1.1 RecName: Full=Transcription factor MYB44; AltName: Full=Myb-related protein 44; Short=AtMYB44; AltName: Full=Myb-related protein R1; Short=AtMYBR1 && PF00249:Myb-like DNA-binding domain	Chr12	1356134	1358815	-	1206
MD07G1024500.v1.1	-1.768588	3.64E-06	- && Q4PIP8.2 RecName: Full=Protein PNS1 && PF04515:Plasma-membrane choline transporter	Chr07	2046172	2049827	-	1764
MD13G1101100.v1.1	-2.829596	4.24E-06	- && Q9SAD4.1 RecName: Full=Ethylene-responsive transcription factor ESR1; AltName: Full=Protein DORNROSCHEN; AltName: Full=Protein ENHANCER OF SHOOT REGENERATION 1 && PF00847:AP2 domain	Chr13	7165695	7166993	-	1299
MD02G1112000.v1.1	-3.151904	4.49E-06	- && Q9SD00.1 RecName: Full=Monosaccharide-sensing protein 3; AltName: Full=Sugar transporter MSSP3 && PF00083:Sugar (and other) transporter	Chr02	9123190	9125466	+	213
MD14G1011900.v1.1	-4.211312	4.77E-06	- && - && -	Chr14	1129546	1131460	-	393
MD06G1157900.v1.1	-1.794767	4.85E-06	- && Q9SWE5.1 RecName: Full=Phosphopantothenoylcysteine decarboxylase; Short=PPCDC; AltName: Full=AtCoaC1; AltName: Full=Halotolerance protein Hal3a; Short=AtHal3a && PF02441:Flavoprotein	Chr06	29965592	29968791	-	669
MD10G1079700.v1.1	-1.63369	4.91E-06	- && Q8VZ80.2 RecName: Full=Polyol transporter 5; AltName: Full=Protein POLYOL TRANSPORTER 5; Short=AtPLT5; AltName: Full=Sugar-proton symporter PLT5 && PF00083:Sugar (and other) transporter	Chr10	11592693	11595756	-	1581
MD04G1198000.v1.1	-2.690533	5.05E-06	- && Q9M9W7.1 RecName: Full=Putative pectinesterase/pectinesterase inhibitor 22; Includes: RecName: Full=Pectinesterase inhibitor 22; AltName: Full=Pectin methylesterase inhibitor 22; Includes: RecName: Full=Pectinesterase 22; Short=PE 22; AltName: Full=Pectin methylesterase 22; Short=AtPME22; Flags: Precursor && PF04043:Plant invertase/pectin methylesterase inhibitor|PF01095:Pectinesterase	Chr04	28574369	28576256	+	1608
MD03G1013200.v1.1	-1.732291	6.28E-06	- && Q42578.1 RecName: Full=Peroxidase 53; Short=Atperox P53; AltName: Full=ATPA2; Flags: Precursor && PF00141:Peroxidase	Chr03	1039521	1041488	-	1056
MD00G1015700.v1.1	-4.89614	7.54E-06	- && Q50228.1 RecName: Full=Formamidase; AltName: Full=Formamide amidohydrolase && PF03069:Acetamidase/Formamidase family	Chr00	2247381	2249286	-	603
MD10G1025300.v1.1	-2.757787	7.63E-06	- && Q6NMB7.1 RecName: Full=WAT1-related protein At1g43650 && PF00892:EamA-like transporter family	Chr10	3178070	3181436	+	1059
MD17G1063800.v1.1	-2.861371	7.91E-06	- && - && -	Chr17	5194960	5196431	+	1452
MD01G1143600.v1.1	-1.233365	1.04E-05	- && Q9M052.1 RecName: Full=UDP-glycosyltransferase 76F1 && PF00201:UDP-glucoronosyl and UDP-glucosyl transferase	Chr01	25391910	25393791	+	1368
MD03G1029900.v1.1	-1.100712	1.05E-05	- && Q9FG72.1 RecName: Full=Oligopeptide transporter 1; Short=AtOPT1 && PF03169:OPT oligopeptide transporter protein	Chr03	2440288	2444998	+	2253
novel.104	4.2450986	1.17E-05	PF02892:BED zinc finger	Chr00	44453211	44455057	+	975
MD07G1230500.v1.1	-1.76938	1.21E-05	- && A7NY33.1 RecName: Full=Peroxidase 4; Flags: Precursor && PF00141:Peroxidase	Chr07	30489128	30490950	-	1011
MD09G1007400.v1.1	-2.411021	1.38E-05	- && Q6K609.1 RecName: Full=Glutaredoxin-C3; AltName: Full=Protein ROXY 2 && PF00462:Glutaredoxin	Chr09	524570	524950	-	381
MD05G1254200.v1.1	-1.890638	1.45E-05	- && O81865.1 RecName: Full=Protein PHLOEM PROTEIN 2-LIKE A1; Short=AtPP2-A1 && PF14299:Phloem protein 2	Chr05	38480134	38481743	-	867
novel.4123	-1.099648	1.47E-05	PF02458:Transferase family	Chr16	7663900	7671921	+	2462

MD02G1094500.v1.1	-1.5164	2.07E-05	- && Q9CAZ7.1 RecName: Full=Protein STRICTOSIDINE SYNTHASE-LIKE 5; Short=AtSSL5; AltName: Full=Protein YELLOW-LEAF-SPECIFIC GENE 2; Flags: Precursor && PF03088:Strictosidine synthase	Chr02	7493023	7497282	+	1074
MD12G1139700.v1.1	-1.288834	2.20E-05	- && Q9M353.1 RecName: Full=Cation/H(+) antiporter 20; AltName: Full=Protein CATION/H+ EXCHANGER 20; Short=AtCHX20 && PF00999:Sodium/hydrogen exchanger family	Chr12	21771600	21778502	-	2538
novel.3941	-1.807281	2.45E-05	PF13855:Leucine rich repeat	Chr15	105962	108559	-	2598
MD01G1211300.v1.1	-1.6766	2.45E-05	- && Q6NW58.2 RecName: Full=Spastin && PF00004:ATPase family associated with various cellular activities (AAA)	Chr01	30486311	30491344	-	2505
MD00G1191600.v1.1	-1.276808	2.85E-05	- && Q9SJI7.1 RecName: Full=Phospholipase A1-IIdelta && PF01764:Lipase (class 3)	Chr00	46484170	46485204	+	1035
MD13G1233500.v1.1	-3.46273	3.22E-05	- && - && -	Chr13	23317934	23320124	-	234
MD06G1030600.v1.1	-1.690845	3.23E-05	- && Q9C5Y2.2 RecName: Full=Ent-kaurenoic acid oxidase 2; Short=AtKAO2; AltName: Full=Cytochrome P450 88A4 && PF00067:Cytochrome P450	Chr06	3652336	3655938	+	1482
MD10G1310600.v1.1	-3.840611	3.25E-05	- && P0CV94.1 RecName: Full=(3S,6E)-nerolidol synthase 1; Short=FaNES1 && PF01397:Terpene synthase, N-terminal domain|PF03936:Terpene synthase family, metal binding domain	Chr10	39558786	39561964	-	1605
MD00G1137700.v1.1	-1.592573	3.46E-05	- && Q41350.1 RecName: Full=Osmotin-like protein; Flags: Precursor && PF00314:Thaumatin family	Chr00	29833805	29834669	+	843
MD17G1014700.v1.1	-1.652545	3.56E-05	- && O24164.1 RecName: Full=Protoporphyrinogen oxidase, mitochondrial; AltName: Full=PX-2; AltName: Full=Protoporphyrinogen IX oxidase isozyme II; Short=PPO II; Short=PPX II && PF01593:Flavin containing amine oxidoreductase	Chr17	1213442	1218261	-	1512
MD08G1066400.v1.1	-1.295213	3.88E-05	- && Q8VZH2.1 RecName: Full=Aminopeptidase M1; AltName: Full=Alpha-aminoacylpeptide hydrolase && PF01433:Peptidase family M1|PF11838:ERAP1-like C-terminal domain	Chr08	5312293	5317663	-	2661
MD02G1291000.v1.1	-3.166305	4.05E-05	- && P51074.2 RecName: Full=Annexin-like protein RJ4 && PF00191:Annexin	Chr02	34675067	34677939	+	942
MD08G1066500.v1.1	-1.555764	4.54E-05	- && Q8VZH2.1 RecName: Full=Aminopeptidase M1; AltName: Full=Alpha-aminoacylpeptide hydrolase && PF11838:ERAP1-like C-terminal domain|PF01433:Peptidase family M1	Chr08	5329121	5335519	+	2667
MD06G1135400.v1.1	-1.503337	4.71E-05	- && - && -	Chr06	28042412	28050535	+	5115
MD06G1146700.v1.1	-1.057775	4.77E-05	- && H2DH17.1 RecName: Full=Cytochrome P450 CYP749A22; AltName: Full=Cytochrome P450 CYP749A20 [Panax ginseng] && PF00067:Cytochrome P450	Chr06	28918899	28921752	-	1554
MD07G1043300.v1.1	-1.625393	4.80E-05	- && Q9FL41.1 RecName: Full=WAT1-related protein At5g07050 && PF00892:EamA-like transporter family	Chr07	3637099	3639341	-	1179
MD09G1085900.v1.1	-2.318634	5.02E-05	- && Q9VR91.3 RecName: Full=Probable E3 ubiquitin-protein ligase HERC2; AltName: Full=HECT domain and RCC1-like domain-containing protein 2; AltName: Full=HECT-type E3 ubiquitin transferase HERC2 && PF00415:Regulator of chromosome condensation (RCC1) repeat|PF00244:14-3-3 protein	Chr09	6114539	6120320	-	1533
MD02G1304400.v1.1	-1.371089	5.02E-05	- && Q9S9N9.1 RecName: Full=Cinnamoyl-CoA reductase 1; Short=AtCCR1; AltName: Full=Protein IRREGULAR XYLEM 4 && PF01370:NAD dependent epimerase/dehydratase family	Chr02	35802895	35806712	+	981
MD12G1100600.v1.1	-1.503043	5.14E-05	- && Q9LZR0.2 RecName: Full=Putative homeobox-leucine zipper protein ATHB-51; AltName: Full=HD-ZIP protein ATHB-51; AltName:	Chr12	15668459	15669906	+	696

			Full=Homeodomain transcription factor ATHB-51 && PF02183:Homeobox associated leucine zipper|PF00046:Homeobox domain					
MD03G1090600.v1.1	-4.446244	5.99E-05	- && Q9SD62.1 RecName: Full=Putative receptor-like protein kinase At3g47110; Flags: Precursor && PF07714:Protein tyrosine kinase|PF08263:Leucine rich repeat N-terminal domain	Chr03	7446903	7450312	+	3072
MD03G1288000.v1.1	-1.222409	6.88E-05	- && Q9ASU7.1 RecName: Full=Peter Pan-like protein && PF04427:Brix domain	Chr03	36680075	36682220	-	1032
MD07G1215900.v1.1	-1.115837	7.87E-05	- && Q9FEL7.1 RecName: Full=Auxin transporter-like protein 2; AltName: Full=AUX1-like protein 2; AltName: Full=MtLAX2 && PF01490:Transmembrane amino acid transporter protein	Chr07	29315572	29319808	+	1470
MD15G1434400.v1.1	-2.982626	8.26E-05	- && Q9C5K8.1 RecName: Full=Protein TIFY 3B; AltName: Full=Jasmonate ZIM domain-containing protein 12 && PF09425:Divergent CCT motif|PF06200:tify domain	Chr15	53470255	53474071	+	810
MD15G1406800.v1.1	-1.9856	8.59E-05	- && - && -	Chr15	50718216	50718638	-	423
MD14G1128000.v1.1	-1.149217	8.92E-05	- && Q42059.2 RecName: Full=Endoglucanase 6; AltName: Full=Endo-1,4-beta glucanase 6; Flags: Precursor && PF09478:Carbohydrate binding domain CBM49|PF00759:Glycosyl hydrolase family 9	Chr14	20435456	20440446	+	1887
MD16G1205300.v1.1	-1.238739	0.000108577	- && Q84LM4.1 RecName: Full=Acylamino-acid-releasing enzyme; Short=AARE; AltName: Full=Oxidized protein hydrolase; Short=OPH && PF00326:Prolyl oligopeptidase family	Chr16	18929355	18936196	-	2463
MD11G1009700.v1.1	-1.425726	0.00012305	- && Q9LHN7.1 RecName: Full=Probable polyamine transporter At3g13620 && PF13520:Amino acid permease	Chr11	769877	771790	-	1470
MD05G1119300.v1.1	-1.444295	0.000128533	- && F4I9E1.1 RecName: Full=Protein NUCLEAR FUSION DEFECTIVE 4 && PF06813:Nodulin-like	Chr05	24041678	24047281	-	1851
novel.1687	-2.200736	0.00013332	-	Chr07	2831306	2832694	+	470
MD04G1021200.v1.1	-1.649302	0.000161349	- && - && PF12657:Transcription factor IIIC subunit delta N-term	Chr04	2497771	2502701	-	2571
MD08G1010700.v1.1	-2.056207	0.000167487	- && Q9SYQ8.3 RecName: Full=Receptor protein kinase CLAVATA1; Flags: Precursor && PF08263:Leucine rich repeat N-terminal domain|PF07714:Protein tyrosine kinase	Chr08	801231	804489	+	2922
MD11G1111100.v1.1	-1.44689	0.000211422	- && Q9M2U1.2 RecName: Full=Dof zinc finger protein DOF3.6; Short=AtDOF3.6; AltName: Full=OBF-binding protein 3 && PF02701:Dof domain, zinc finger	Chr11	9836381	9838175	-	1128
MD08G1078300.v1.1	-1.833169	0.00021535	- && Q00416.2 RecName: Full=Helicase SEN1; AltName: Full=tRNA-splicing endonuclease positive effector && PF13087:AAA domain|PF13086:AAA domain	Chr08	6510393	6515259	+	2724
MD10G1051100.v1.1	-1.710538	0.000237362	- && Q7XJE6.1 RecName: Full=Metacaspase-1; Short=AtMC1; AltName: Full=Metacaspase 1b; Short=AtMCP1b; AltName: Full=Protein LSD ONE LIKE 3 && PF00656:Caspase domain	Chr10	6749111	6754200	-	1284
MD17G1244800.v1.1	-1.677153	0.000265841	- && Q9M884.1 RecName: Full=Mannose-6-phosphate isomerase 1; AltName: Full=Phosphohexomutase 1; AltName: Full=Phosphomannose isomerase 1; Short=PMI1; AltName: Full=Protein MATERNAL EFFECT EMBRYO ARREST 31 && PF01238:Phosphomannose isomerase type I	Chr17	29320749	29323928	-	1326
MD08G1013100.v1.1	-2.135919	0.00026795	- && - && -	Chr08	967088	967495	+	408
MD17G1246800.v1.1	-5.006329	0.000272702	- && O22765.2 RecName: Full=Tryptophan synthase alpha chain; AltName: Full=Indole synthase; AltName: Full=Indole-3-glycerol-phosphate lyase && PF00290:Tryptophan synthase alpha chain	Chr17	29517547	29519040	-	1164
novel.1262	-2.141645	0.000281383	PF01565:FAD binding domain|PF08031:Berberine and berberine like	Chr05	40368253	40371161	+	2350

MD16G1281100.v1.1	-1.326777	0.000311735	- && - && -	Chr16	38285712	38286967	+	534
MD02G1149600.v1.1	-1.414617	0.000340219	- && - && PF01925:Sulfite exporter TauE/SafE	Chr02	12317148	12320639	-	1428
MD11G1168000.v1.1	-1.806282	0.000365934	- && Q9SEA0.1 RecName: Full=Capsanthin/capsorubin synthase, chromoplastic; Flags: Precursor && PF05834:Lycopene cyclase protein	Chr11	17970776	17971715	+	879
MD01G1087300.v1.1	-2.440229	0.00043385	- && Q9MAM1.2 RecName: Full=CBL-interacting serine/threonine-protein kinase 9; AltName: Full=SNF1-related kinase 3.12; AltName: Full=SOS2-like protein kinase PKS6 && PF03822:NAF domain|PF00069:Protein kinase domain	Chr01	19657705	19663688	+	1428
MD02G1196800.v1.1	-1.406312	0.000435713	- && Q84WS0.1 RecName: Full=Subtilisin-like protease SBT1.1; AltName: Full=Subtilase subfamily 1 member 1; Short=AtSBT1.1; Flags: Precursor && PF00082:Subtilase family|PF02225:PA domain|PF05922:Peptidase inhibitor I9	Chr02	18929017	18931876	-	2256
MD05G1238400.v1.1	-1.796631	0.000448333	- && Q9SAK5.2 RecName: Full=Myb family transcription factor APL; Short=AtAPL; AltName: Full=Protein ALTERED PHLOEM DEVELOPMENT; AltName: Full=Protein FE; AltName: Full=Protein PHOSPHATE STARVATION RESPONSE 2; Short=AtPHR2; AltName: Full=Protein PHR1-LIKE 14; AltName: Full=Protein WOODY && PF14379:MYB-CC type transfactor, LHEQLE motif|PF00249:Myb-like DNA-binding domain	Chr05	36957022	36959513	+	1101
MD13G1205800.v1.1	-1.961916	0.000481441	- && - && PF14576:Sieve element occlusion N-terminus	Chr13	18658544	18659913	+	474
MD10G1202800.v1.1	-4.724987	0.000555952	- && O81832.4 RecName: Full=G-type lectin S-receptor-like serine/threonine-protein kinase At4g27290; Flags: Precursor && PF07714:Protein tyrosine kinase|PF01453:D-mannose binding lectin|PF00954:S-locus glycoprotein domain|PF08276:PAN-like domain	Chr10	30039754	30043166	-	2502
MD16G1015600.v1.1	-1.546393	0.000555952	- && Q9XID3.1 RecName: Full=G-type lectin S-receptor-like serine/threonine-protein kinase At1g34300; Flags: Precursor && PF00954:S-locus glycoprotein domain|PF00069:Protein kinase domain|PF01453:D-mannose binding lectin	Chr16	1210771	1213170	+	2400
MD12G1026200.v1.1	-1.438492	0.000579051	- && Q9XIH7.1 RecName: Full=Putative polyol transporter 1 && PF00083:Sugar (and other) transporter	Chr12	2901374	2903922	-	1581
MD11G1307200.v1.1	-1.126373	0.000591832	- && O81301.1 RecName: Full=Probable pectinesterase/pectinesterase inhibitor 40; Includes: RecName: Full=Pectinesterase inhibitor 40; AltName: Full=Pectin methylesterase inhibitor 40; Includes: RecName: Full=Pectinesterase 40; Short=PE 40; AltName: Full=Pectin methylesterase 40; Short=AtPME40; Flags: Precursor && PF04043:Plant invertase/pectin methylesterase inhibitor|PF01095:Pectinesterase	Chr11	42133236	42138138	-	1740
MD11G1156200.v1.1	-1.190498	0.000598459	- && Q9FNI7.1 RecName: Full=Glucomannan 4-beta-mannosyltransferase 2; AltName: Full=Cellulose synthase-like protein A2; Short=AtCslA2; AltName: Full=Glucomannan synthase; AltName: Full=Mannan synthase 2 && PF13641:Glycosyltransferase like family 2	Chr11	15012290	15017935	+	1650
MD06G1185200.v1.1	-1.229672	0.000630051	- && Q9SSG3.2 RecName: Full=HIPL1 protein; Flags: Precursor && PF07995:Glucose / Sorbosone dehydrogenase	Chr06	32312645	32316327	-	2082
novel.3162	-1.106893	0.000731581	-	Chr12	27072745	27074735	+	944
MD11G1012300.v1.1	-2.771505	0.000918726	- && - && PF04819:Family of unknown function (DUF716)	Chr11	886298	887248	-	951
MD03G1106600.v1.1	-3.155147	0.000935083	- && Q9FJD5.1 RecName: Full=Laccase-17; AltName: Full=Benzenediol:oxygen oxidoreductase 17; AltName: Full=Diphenol	Chr03	9179028	9181970	+	1740

			oxidase 17; AltName: Full=Urishiol oxidase 17; Flags: Precursor && PF00394:Multicopper oxidase|PF07731:Multicopper oxidase|PF07732:Multicopper oxidase					
MD01G1115200.v1.1	-1.116465	0.000955666	- && Q7SXR3.2 RecName: Full=Macrophage erythroblast attacher && PF10607:CTLH/CRA C-terminal to LisH motif domain|PF13445:RING-type zinc-finger	Chr01	22935644	22940010	+	1248
MD02G1281400.v1.1	-6.414789	0.00106665	- && Q623T0.1 RecName: Full=2-oxoglutarate dehydrogenase, mitochondrial; AltName: Full=2-oxoglutarate dehydrogenase complex component E1; Short=OGDC-E1; AltName: Full=Alpha-ketoglutarate dehydrogenase; Flags: Precursor && PF16870:2-oxoglutarate dehydrogenase C-terminal	Chr02	33724739	33726075	-	522
MD02G1085400.v1.1	-3.295134	0.001079364	- && - && PF05553:Cotton fibre expressed protein	Chr02	6695543	6697258	+	1716
MD09G1070100.v1.1	-1.655176	0.001097025	- && Q8GXJ4.2 RecName: Full=Glutamate receptor 3.4; Short=AtGLR4; AltName: Full=Ligand-gated ion channel 3.4; Flags: Precursor && PF00497:Bacterial extracellular solute-binding proteins, family 3|PF01094:Receptor family ligand binding region|PF00060:Ligand-gated ion channel	Chr09	4822947	4829801	-	2964
MD13G1096600.v1.1	-1.416104	0.001125622	- && Q9CAL2.1 RecName: Full=Cysteine-rich receptor-like protein kinase 3; Short=Cysteine-rich RLK3; Flags: Precursor && PF01657:Salt stress response/antifungal|PF00069:Protein kinase domain	Chr13	6833836	6838261	-	1947
MD16G1095500.v1.1	-1.72187	0.001127657	- && Q94AJ5.1 RecName: Full=Probable polygalacturonase At1g80170; Short=PG; AltName: Full=Pectinase At1g80170; Flags: Precursor && PF13041:PPR repeat family|PF00295:Glycosyl hydrolases family 28	Chr16	6606828	6612450	-	3399
MD10G1014900.v1.1	-5.602176	0.001157169	- && - && -	Chr10	2013344	2013901	-	558
MD07G1237300.v1.1	-1.165329	0.001292445	- && Q9LVM0.1 RecName: Full=Probable inactive receptor kinase At5g58300; Flags: Precursor && PF07714:Protein tyrosine kinase|PF08263:Leucine rich repeat N-terminal domain	Chr07	30975210	30978030	-	1908
novel.4061	-2.198287	0.001312444	-	Chr15	42974938	42976028	-	679
MD00G1084300.v1.1	-2.124849	0.0013334	- && - && -	Chr00	16842495	16848839	+	1797
novel.2787	-1.328114	0.001340014	-	Chr11	6674387	6676982	+	2399
novel.4357	-1.286613	0.001349142	-	Chr17	10212027	10213974	+	1948
MD03G1110000.v1.1	-1.01355	0.00135085	- && Q84TE9.1 RecName: Full=Dof zinc finger protein DOF5.3; Short=AtDOF5.3 && PF02701:Dof domain, zinc finger	Chr03	9569797	9572071	+	873
MD02G1029900.v1.1	-2.892683	0.001392816	- && Q84XI3.1 RecName: Full=Equilibrative nucleotide transporter 8; Short=AtENT8; AltName: Full=Nucleoside transporter ENT8 && PF01733:Nucleoside transporter	Chr02	2314928	2316448	+	1200
MD01G1059600.v1.1	-1.184553	0.001394614	- && P93604.1 RecName: Full=Rust resistance kinase Lr10; AltName: Full=Probable receptor-like serine/threonine-protein kinase LRK10; Flags: Precursor && PF00069:Protein kinase domain	Chr01	16349629	16351638	-	1818
MD15G1189400.v1.1	-1.129737	0.001437453	- && Q10QA5.1 RecName: Full=Strigolactone esterase D14; AltName: Full=Protein DWARF 14; AltName: Full=Protein DWARF 88; AltName: Full=Protein HIGH-TILLERING DWARF 2 && PF12695:Alpha/beta hydrolase family	Chr15	14944613	14946567	+	858
MD06G1033100.v1.1	-1.834462	0.001483772	- && Q9C5C4.1 RecName: Full=Acetylornithine deacetylase; AltName: Full=N-acetylornithinase; Short=AO; Short=Acetylornithinase; Short=NAO	Chr06	3960322	3963717	-	1320

			&& PF01546:Peptidase family M20/M25/M40|PF07687:Peptidase dimerisation domain					
MD15G1023100.v1.1	-1.562505	0.001484941	- && Q9SFZ3.2 RecName: Full=Transcription factor bHLH110; AltName: Full=Basic helix-loop-helix protein 110; Short=AtbHLH110; Short=bHLH 110; AltName: Full=Transcription factor EN 59; AltName: Full=bHLH transcription factor bHLH110 && -	Chr15	1346056	1351663	+	1404
MD14G1149800.v1.1	-1.232302	0.001525779	- && - && PF00536:SAM domain (Sterile alpha motif)	Chr14	24284651	24285577	+	927
MD05G1248500.v1.1	-1.312566	0.001715683	- && - && PF04640:PLATZ transcription factor	Chr05	37994133	37997028	+	765
MD09G1253100.v1.1	-1.033348	0.001804008	- && Q9M884.1 RecName: Full=Mannose-6-phosphate isomerase 1; AltName: Full=Phosphohexomutase 1; AltName: Full=Phosphomannose isomerase 1; Short=PMI1; AltName: Full=Protein MATERNAL EFFECT EMBRYO ARREST 31 && PF01238:Phosphomannose isomerase type I	Chr09	32414835	32418809	-	1329
MD06G1202700.v1.1	-1.260924	0.002079937	- && Q9FN02.1 RecName: Full=Serine/threonine-protein phosphatase 7 && PF00149:Calcineurin-like phosphoesterase	Chr06	33587486	33592104	-	2115
MD12G1048700.v1.1	-3.039343	0.002088953	- && Q84WU2.1 RecName: Full=Ubiquitin carboxyl-terminal hydrolase 13; AltName: Full=Deubiquitinating enzyme 13; Short=AtUBP13; AltName: Full=Ubiquitin thioesterase 13; AltName: Full=Ubiquitin-specific-processing protease 13 && PF12436:ICP0-binding domain of Ubiquitin-specific protease 7|PF14533:Ubiquitin-specific protease C-terminal	Chr12	5534702	5540043	-	2244
MD13G1143500.v1.1	-1.431781	0.002214664	- && Q9FPT1.2 RecName: Full=Ubiquitin carboxyl-terminal hydrolase 12; AltName: Full=Deubiquitinating enzyme 12; Short=AtUBP12; AltName: Full=Ubiquitin thioesterase 12; AltName: Full=Ubiquitin-specific-processing protease 12 && PF00917:MATH domain	Chr13	11163848	11170211	-	1425
novel.535	-1.167145	0.00221614	PF14226:non-haem dioxygenase in morphine synthesis N-terminal|PF00294:pfkB family carbohydrate kinase	Chr02	5404658	5407295	-	2638
MD09G1187300.v1.1	-3.108121	0.002448046	- && - && PF05553:Cotton fibre expressed protein	Chr09	16328595	16330379	-	1785
MD17G1286900.v1.1	-3.93095	0.002463771	- && Q7XNX6.2 RecName: Full=Sucrose synthase 7; Short=OsSUS7; AltName: Full=Sucrose-UDP glucosyltransferase 7 && PF00862:Sucrose synthase	Chr17	34650839	34654753	-	2034
MD05G1047800.v1.1	-1.550099	0.002504078	- && F4IFF3.1 RecName: Full=Probable DNA helicase MCM9; AltName: Full=Minichromosome maintenance 9; Short=AtMCM9 && PF00493:MCM2/3/5 family	Chr05	8123939	8142120	+	1998
MD02G1318000.v1.1	-1.216777	0.002538344	- && - && PF04438:HIT zinc finger	Chr02	37251101	37254242	-	1239
MD16G1027400.v1.1	-1.726969	0.002565602	- && - && -	Chr16	1931059	1932757	-	369
MD01G1092000.v1.1	2.5822441	0.002664137	- && - && -	Chr01	20688870	20689906	-	450
MD01G1112800.v1.1	-3.145115	0.002695664	- && - && -	Chr01	22688525	22690626	-	213
MD11G1230400.v1.1	-1.196508	0.002697784	- && Q9LSQ4.1 RecName: Full=Indole-3-acetic acid-amido synthetase GH3.6; AltName: Full=Auxin-responsive GH3-like protein 6; Short=AtGH3-6; AltName: Full=Protein DWARF IN LIGHT 1; Short=DFL-1 && PF03321:GH3 auxin-responsive promoter	Chr11	33501665	33504059	-	1845
MD15G1103400.v1.1	-1.374479	0.002701267	- && Q9FUY2.2 RecName: Full=Transcriptional corepressor LEUNIG; AltName: Full=Protein ROTUNDA2 && -	Chr15	7294069	7296915	-	1008
MD14G1135300.v1.1	-1.14927	0.00299847	- && Q84JQ8.1 RecName: Full=Dof zinc finger protein DOF1.8; Short=AtDOF1.8 && PF02701:Dof domain, zinc finger	Chr14	21507409	21509559	+	948

MD06G1153900.v1.1	-2.605103	0.003056667	- && - && PF10193:Telomere length regulation protein	Chr06	29723200	29727343	-	849
MD05G1336900.v1.1	-4.310134	0.003069083	- && - && -	Chr05	45868370	45871670	-	231
MD15G1139800.v1.1	-1.682895	0.003117832	- && Q84KJ6.1 RecName: Full=Ammonium transporter 3 member 1; Short=OsAMT3;1 && PF00909:Ammonium Transporter Family	Chr15	10278806	10283836	+	1470
MD11G1043500.v1.1	-1.177366	0.003125455	- && Q9LRR4.1 RecName: Full=Putative disease resistance RPP13-like protein 1 && PF00931:NB-ARC domain	Chr11	3724133	3734235	-	4098
MD04G1167200.v1.1	-2.090072	0.003223986	- && Q9C7S5.1 RecName: Full=Tyrosine-sulfated glycopeptide receptor 1; AltName: Full=PSY1 receptor && PF08263:Leucine rich repeat N-terminal domain	Chr04	25747284	25749944	-	2364
MD06G1238700.v1.1	-1.978623	0.003283083	- && - && PF14364:Domain of unknown function (DUF4408)|PF05553:Cotton fibre expressed protein	Chr06	36936139	36937218	-	1080
novel.3994	-1.576011	0.003324962	-	Chr15	13117637	13118647	-	615
MD10G1052000.v1.1	-1.601548	0.003336271	- && Q8RXE8.1 RecName: Full=Probable sodium/metabolite cotransporter BASS3, chloroplastic; AltName: Full=Bile acid transporter 3; AltName: Full=Bile acid-sodium symporter family protein 3; Flags: Precursor && PF01758:Sodium Bile acid symporter family	Chr10	6898902	6899967	-	294
MD03G1130600.v1.1	-1.309665	0.003396904	- && Q9SJK3.2 RecName: Full=Protein argonaute 5 && PF16488:Argonaute linker 2 domain|PF16487:Mid domain of argonaute|PF02171:Piwi domain|PF08699:Argonaute linker 1 domain|PF16486:N-terminal domain of argonaute|PF02170:PAZ domain	Chr03	12839498	12846674	+	3057
MD11G1254900.v1.1	-2.428557	0.003440623	- && Q9LXA5.1 RecName: Full=L-type lectin-domain containing receptor kinase IX.1; Short=LecRK-IX.1; Flags: Precursor && PF00069:Protein kinase domain|PF00139:Legume lectin domain	Chr11	36764832	36766928	-	2097
MD11G1180200.v1.1	-2.056251	0.00347424	- && Q8L3Z8.1 RecName: Full=Protein FIZZY-RELATED 2; AltName: Full=Cell cycle switch protein CCS52A1 && -	Chr11	21893460	21894230	-	414
MD02G1111900.v1.1	-4.293824	0.003549921	- && - && -	Chr02	9122134	9122812	+	375
MD04G1181800.v1.1	-1.385449	0.003611878	- && Q9LT96.1 RecName: Full=Probable leucine-rich repeat receptor-like protein kinase At5g49770; Flags: Precursor && PF07714:Protein tyrosine kinase	Chr04	27273902	27278398	-	2499
MD16G1013000.v1.1	-1.165757	0.003762481	- && - && -	Chr16	999362	1000839	+	729
MD14G1204400.v1.1	-1.002989	0.003783766	- && Q9FLR0.1 RecName: Full=Protein NEN1; AltName: Full=NAC45/NAC86-dependent exonuclease-domain protein 1 && PF00929:Exonuclease	Chr14	29240725	29243501	+	1521
MD09G1041500.v1.1	-1.84299	0.003925261	- && Q9LHD1.1 RecName: Full=ABC transporter B family member 15; Short=ABC transporter ABCB.15; Short=AtABCB15; AltName: Full=Multidrug resistance protein 13; AltName: Full=P-glycoprotein 15 && PF00664:ABC transporter transmembrane region|PF00005:ABC transporter|PF07673:Protein of unknown function (DUF1602)	Chr09	2676919	2684418	-	3771
MD02G1310600.v1.1	-1.693175	0.004164632	- && - && -	Chr02	36588611	36589456	-	150
MD03G1204900.v1.1	-2.908191	0.004307282	- && Q6WG30.2 RecName: Full=Taxadiene 5-alpha hydroxylase && PF00067:Cytochrome P450	Chr03	28116773	28117924	+	996
MD14G1188700.v1.1	-1.546959	0.004699313	- && Q9SU72.1 RecName: Full=Protein EDS1; AltName: Full=Enhanced disease susceptibility 1 && PF01764:Lipase (class 3)	Chr14	28038549	28041908	+	1857
MD07G1077600.v1.1	-1.695249	0.004727477	- && Q9LF64.1 RecName: Full=RING-H2 finger protein ATL52; AltName: Full=RING-type E3 ubiquitin transferase ATL52 && PF13639:Ring finger domain	Chr07	7408477	7409562	-	1086

MD14G1156300.v1.1	-1.680457	0.005015555	- && A8FMW6.1 RecName: Full=Chaperone protein DnaJ && PF11926:Domain of unknown function (DUF3444)|PF00226:DnaJ domain	Chr14	25069510	25072575	+	2661
MD16G1175800.v1.1	-1.47619	0.00512301	- && Q56XP4.2 RecName: Full=Sodium/hydrogen exchanger 2; AltName: Full=Na(+)/H(+) exchanger 2; Short=NHE-2 && PF00999:Sodium/hydrogen exchanger family	Chr16	14911647	14916135	+	1656
MD17G1116800.v1.1	-1.068167	0.005190169	- && O19071.1 RecName: Full=Alpha-1,6-mannosyl-glycoprotein 2-beta-N-acetylglucosaminyltransferase; AltName: Full=Beta-1,2-N-acetylglucosaminyltransferase II; AltName: Full=GlcNAc-T II; Short=GNT-II; AltName: Full=Mannoside acetylglucosaminyltransferase 2; AltName: Full=N-glycosyl-oligosaccharide-glycoprotein N-acetylglucosaminyltransferase II && PF05060:N-acetylglucosaminyltransferase II (MGAT2)	Chr17	10064196	10065479	-	1284
MD01G1124800.v1.1	-2.202387	0.005441605	- && Q9M1Y3.1 RecName: Full=Ankyrin repeat protein SKIP35; AltName: Full=SKP1-interacting partner 35 && -	Chr01	23762549	23764156	-	1608
MD01G1104700.v1.1	-3.156326	0.005636692	- && - && PF01490:Transmembrane amino acid transporter protein	Chr01	21790048	21793817	+	1083
MD08G1133500.v1.1	-2.420323	0.005725406	- && Q6YXX9.1 RecName: Full=Guanine nucleotide-binding protein subunit gamma 2; AltName: Full=Ggamma-subunit 2; AltName: Full=Heterotrimeric G protein gamma-subunit 2 >A2X0N9.1 RecName: Full=Guanine nucleotide-binding protein subunit gamma 2; AltName: Full=Ggamma-subunit 2; AltName: Full=Heterotrimeric G protein gamma-subunit 2 && PF00631:GGL domain	Chr08	12671549	12672770	+	393
MD13G1112700.v1.1	-1.962906	0.00608453	- && Q8VWZ7.1 RecName: Full=Geraniol 8-hydroxylase; AltName: Full=Cytochrome P450 76B6; AltName: Full=Geraniol 10-hydroxylase; Short=CrG10H && PF00067:Cytochrome P450	Chr13	8183162	8185070	+	1515
MD08G1207200.v1.1	-1.429999	0.00624716	- && - && PF00092:von Willebrand factor type A domain|PF17123:RING-like zinc finger	Chr08	26922288	26925097	-	2247
MD16G1242500.v1.1	-2.022549	0.006402906	- && Q9C9W0.1 RecName: Full=ABC transporter I family member 17; Short=ABC transporter ABCI.17; Short=AtABCI17; AltName: Full=MRP-related protein 1; AltName: Full=Non-intrinsic ABC protein 3 && PF00005:ABC transporter	Chr16	26241038	26243961	+	621
MD03G1205100.v1.1	-1.080517	0.006462076	- && Q50EK1.1 RecName: Full=Cytochrome P450 716B1; AltName: Full=Cytochrome P450 CYPA1 && PF00067:Cytochrome P450	Chr03	28126631	28129094	+	1428
MD06G1157700.v1.1	-1.333315	0.006546993	- && - && PF13920:Zinc finger, C3HC4 type (RING finger)	Chr06	29957963	29960894	+	762
MD07G1217100.v1.1	-1.46967	0.006639608	- && - && -	Chr07	29493979	29495697	-	276
MD16G1101200.v1.1	-1.01651	0.006696051	- && Q9C9V6.1 RecName: Full=BTB/POZ domain-containing protein At1g67900 && PF03000:NPH3 family	Chr16	7020075	7024024	+	1899
MD02G1105500.v1.1	-3.324474	0.00670277	- && Q9M088.1 RecName: Full=Glucan endo-1,3-beta-glucosidase 5; AltName: Full=(1->3)-beta-glucan endohydrolase 5; Short=(1->3)-beta-glucanase 5; AltName: Full=Beta-1,3-endoglucanase 5; Short=Beta-1,3-glucanase 5; Flags: Precursor && PF00332:Glycosyl hydrolases family 17|PF07983:X8 domain	Chr02	8551707	8553467	-	1491
MD04G1184700.v1.1	-2.091368	0.006776753	- && Q9SFF6.1 RecName: Full=Pectin acetylesterase 12; Flags: Precursor && PF03283:Pectinacetylesterase	Chr04	27584295	27587144	+	1224
MD09G1055800.v1.1	-2.279183	0.006963098	- && O64973.2 RecName: Full=Disease resistance protein RPS5; AltName: Full=Resistance to Pseudomonas syringae protein 5; AltName: Full=pNd3/pNd10 && PF13855:Leucine rich repeat|PF00931:NB-ARC domain	Chr09	3700657	3703464	-	2808

MD17G1101100.v1.1	-1.231671	0.006982342	- && - && PF13962:Domain of unknown function|PF14244:gag-polypeptide of LTR copia-type	Chr17	8562251	8569413	-		2361
MD13G1065400.v1.1	-2.543517	0.007090266	- && - && PF03763:Remorin, C-terminal region	Chr13	4541155	4542768	+		783
MD09G1263700.v1.1	-1.756827	0.007090266	- && Q0WNW4.1 RecName: Full=Myosin-binding protein 3 && PF04576:Zein-binding	Chr09	33683707	33687888	-		2670
MD12G1204000.v1.1	-1.415978	0.007176897	- && Q9SIH1.1 RecName: Full=Peptidyl-prolyl cis-trans isomerase CYP18-2; Short=PPIase CYP18-2; AltName: Full=Cyclophilin of 18 kDa 2; AltName: Full=Cyclophilin-18-2 && PF00160:Cyclophilin type peptidyl-prolyl cis-trans isomerase/CLD	Chr12	28477085	28480110	+		492
MD10G1250100.v1.1	-2.732412	0.007257683	- && Q94A40.2 RecName: Full=Coatomer subunit alpha-1; AltName: Full=Alpha-coat protein 1; Short=Alpha-COP 1 && PF06957:Coatomer (COPI) alpha subunit C-terminus	Chr10	34279938	34281142	-		1140
MD02G1151100.v1.1	-1.075978	0.007313172	- && Q9ZU65.1 RecName: Full=Transcription repressor OFP7; AltName: Full=Ovate family protein 7; Short=AtOFP7 && PF04844:Transcriptional repressor, ovate	Chr02	12466155	12467117	+		963
MD13G1189100.v1.1	-1.873011	0.007334814	- && Q94AZ2.2 RecName: Full=Sugar transport protein 13; AltName: Full=Hexose transporter 13; AltName: Full=Multicopy suppressor of snf4 deficiency protein 1 && PF00083:Sugar (and other) transporter	Chr13	16144850	16150276	-		1587
MD08G1205000.v1.1	-2.123836	0.00753515	- && - && PF06045:Rhamnogalacturonate lyase family|PF14683:Polysaccharide lyase family 4, domain III|PF14686:Polysaccharide lyase family 4, domain II	Chr08	26541730	26547707	-		1941
MD17G1131100.v1.1	-4.066079	0.007849367	- && Q9SAB5.1 RecName: Full=Putative F-box/LRR-repeat/kelch-repeat protein At1g11620 && PF00646:F-box domain	Chr17	11560246	11561765	+		918
MD06G1128500.v1.1	-1.393499	0.00808573	- && Q2HXL0.2 RecName: Full=Respiratory burst oxidase homolog protein C; AltName: Full=NADPH oxidase RBOHC; AltName: Full=StRBOHC && PF08030:Ferric reductase NAD binding domain|PF08414:Respiratory burst NADPH oxidase|PF01794:Ferric reductase like transmembrane component|PF08022:FAD-binding domain	Chr06	27087364	27094696	+		2826
MD04G1177000.v1.1	-2.635391	0.008391969	- && Q9C9Z8.1 RecName: Full=V-type proton ATPase subunit E2; Short=V-ATPase subunit E2; AltName: Full=Vacuolar H(+)-ATPase subunit E isoform 2; AltName: Full=Vacuolar proton pump subunit E2 && PF01991:ATP synthase (E/31 kDa) subunit	Chr04	26835265	26837405	+		681
MD10G1203800.v1.1	-1.592924	0.008708294	- && - && PF06203:CCT motif	Chr10	30283788	30286122	-		843
novel.2164	-3.057786	0.008910546	-	Chr08	23926580	23927488	-		815
MD05G1177700.v1.1	-2.369539	0.009458677	- && Q84T03.1 RecName: Full=DEAD-box ATP-dependent RNA helicase 27 && PF13959:Domain of unknown function (DUF4217)	Chr05	30464440	30466308	+		561
MD02G1112600.v1.1	-1.439835	0.010289789	- && - && -	Chr02	9164602	9165474	+		342
MD09G1083800.v1.1	-1.216605	0.010342853	- && - && PF08137:DVL family	Chr09	5929771	5929923	-		153
MD01G1153600.v1.1	-2.262507	0.010374227	- && D4N501.1 RecName: Full=Probable 2-oxoglutarate/Fe(II)-dependent dioxygenase && PF14226:non-haem dioxygenase in morphine synthesis N-terminal|PF03171:2OG-Fe(II) oxygenase superfamily	Chr01	26193698	26196298	-		1086
MD06G1133700.v1.1	-1.061881	0.010706085	- && - && -	Chr06	27809804	27811468	+		1665
MD09G1118500.v1.1	-1.348272	0.010837225	- && - && PF13499:EF-hand domain pair	Chr09	9116148	9116576	-		429
MD08G1203600.v1.1	-1.841886	0.011245705	- && Q40392.1 RecName: Full=TMV resistance protein N && PF00931:NB-ARC domain|PF01582:TIR domain	Chr08	26321687	26325810	-		2931

MD06G1180400.v1.1	-1.748242	0.011527855	- && C0SVV6.1 RecName: Full=Calmodulin-binding protein 60 A && PF07887:Calmodulin binding protein-like	Chr06	31993310	31998987	-	1653
MD09G1116900.v1.1	-1.772485	0.011905766	- && O49636.1 RecName: Full=Mitochondrial pyruvate carrier 4 && PF03650:Uncharacterised protein family (UPF0041)	Chr09	8991364	8993344	+	330
MD12G1136300.v1.1	-1.534299	0.012020928	- && - && PF09713:Plant protein 1589 of unknown function (A_thal_3526)	Chr12	21280051	21281314	-	318
MD08G1005500.v1.1	-1.792218	0.012487602	- && P48147.2 RecName: Full=Prolyl endopeptidase; Short=PE; AltName: Full=Post-proline cleaving enzyme && PF02897:Prolyl oligopeptidase, N-terminal beta-propeller domain|PF00326:Prolyl oligopeptidase family	Chr08	485459	491151	-	2316
MD14G1025800.v1.1	-1.902622	0.012838351	- && - && -	Chr14	2353185	2355199	-	1527
MD04G1144200.v1.1	-2.213902	0.013005079	- && - && PF08571:Yos1-like	Chr04	23322136	23323319	-	318
MD08G1096300.v1.1	-1.498676	0.01301939	- && Q6X7J9.1 RecName: Full=WUSCHEL-related homeobox 4 && PF00046:Homeobox domain	Chr08	8081170	8082937	+	990
MD13G1046500.v1.1	-1.353005	0.013473492	- && - && PF08137:DVL family	Chr13	3222841	3222993	+	153
MD09G1020300.v1.1	1.0772022	0.0140551	- && Q84XV2.1 RecName: Full=Transcription factor GTE1; AltName: Full=Bromodomain-containing protein GTE1; AltName: Full=Protein GLOBAL TRANSCRIPTION FACTOR GROUP E1; AltName: Full=Protein IMBIBITION-INDUCIBLE 1 && PF17035:Bromodomain extra-terminal - transcription regulation	Chr09	1239444	1242840	+	981
MD05G1312400.v1.1	-1.071744	0.014171331	- && Q9LSL6.1 RecName: Full=Dof zinc finger protein DOF5.7; Short=AtDOF5.7 && PF02701:Dof domain, zinc finger	Chr05	44216735	44218220	+	1137
MD04G1008400.v1.1	-3.431192	0.014172653	- && Q75HY5.1 RecName: Full=Zinc finger BED domain-containing protein RICESLEEPER 3; AltName: Full=Transposase-like protein RICESLEEPER 3 && -	Chr04	973854	978396	-	912
MD05G1007300.v1.1	-1.698412	0.014396718	- && - && -	Chr05	1766708	1769419	-	726
MD13G1007400.v1.1	-2.605149	0.014463295	- && Q9LIH7.1 RecName: Full=Protein S-acyltransferase 11; AltName: Full=Probable palmitoyltransferase At3g18620; AltName: Full=Zinc finger DHHC domain-containing protein At3g18620 && PF01529:DHHC palmitoyltransferase	Chr13	470554	471269	+	507
MD08G1135400.v1.1	-2.052993	0.01498016	- && Q9SL95.1 RecName: Full=Molybdate transporter 1; AltName: Full=Sulfate transporter like protein 5.2 && PF16983:Molybdate transporter of MFS superfamily	Chr08	12855713	12857735	+	1395
MD13G1216700.v1.1	-2.413057	0.014991483	- && B5WWZ8.1 RecName: Full=Long-chain-alcohol oxidase FAO1; AltName: Full=Long-chain fatty alcohol oxidase 1 && PF05199:GMC oxidoreductase|PF00732:GMC oxidoreductase	Chr13	20707140	20712396	+	2184
MD04G1161600.v1.1	-2.255163	0.015759819	- && - && PF13855:Leucine rich repeat|PF08263:Leucine rich repeat N-terminal domain	Chr04	25258457	25263867	+	3861
MD05G1241900.v1.1	-1.184854	0.016280079	- && O65440.3 RecName: Full=Leucine-rich repeat receptor-like serine/threonine-protein kinase BAM3; AltName: Full=Protein BARELY ANY MERISTEM 3; Flags: Precursor && PF00069:Protein kinase domain|PF13855:Leucine rich repeat|PF08263:Leucine rich repeat N-terminal domain	Chr05	37501499	37506476	+	2964
MD03G1277000.v1.1	-2.523542	0.016330231	- && Q56XP4.2 RecName: Full=Sodium/hydrogen exchanger 2; AltName: Full=Na(+)/H(+) exchanger 2; Short=NHE-2 && PF00999:Sodium/hydrogen exchanger family	Chr03	35949775	35954850	-	1638
MD16G1104800.v1.1	-3.58198	0.01654797	- && Q9SS87.1 RecName: Full=Protein SIEVE ELEMENT OCCLUSION B; Short=AtSEOb; AltName: Full=Protein SIEVE ELEMENT OCCLUSION-	Chr16	7365969	7368737	+	1749

			RELATED 1; Short=AtSEOR1 && PF14576:Sieve element occlusion N-terminus					
novel.1836	-1.240111	0.017429241	PF00069:Protein kinase domain|PF07714:Protein tyrosine kinase	Chr07	6764626	6766931	-	1671
MD05G1129600.v1.1	-2.688991	0.01839327	- && Q9SX98.1 RecName: Full=Lysine histidine transporter-like 8; AltName: Full=Amino acid transporter-like protein 1 && PF01490:Transmembrane amino acid transporter protein	Chr05	25370086	25375172	-	1482
MD14G1227700.v1.1	-5.584925	0.01862279	- && - && PF13398:Peptidase M50B-like	Chr14	30876999	30878975	-	732
MD17G1097100.v1.1	-2.56073	0.019143126	- && - && -	Chr17	8233140	8233844	+	366
MD11G1126000.v1.1	-3.056648	0.019447946	- && - && -	Chr11	11596169	11598095	-	183
MD01G1041300.v1.1	-1.715296	0.019455029	- && Q6YU51.1 RecName: Full=Probable 1-deoxy-D-xylulose-5-phosphate synthase 2, chloroplastic; Short=1-deoxyxylulose-5-phosphate synthase; Short=DXP synthase; Short=DXPS; Flags: Precursor && PF13292:1-deoxy-D-xylulose-5-phosphate synthase|PF02779:Transketolase, pyrimidine binding domain|PF02780:Transketolase, C-terminal domain	Chr01	13864724	13868894	+	2145
MD16G1160400.v1.1	-1.735196	0.020754031	- && Q40280.3 RecName: Full=Major allergen Mal d 1; AltName: Full=AP15; AltName: Full=Allergen Mal d I; AltName: Allergen=Mal d 1 && PF00407:Pathogenesis-related protein Bet v I family	Chr16	13059984	13061125	-	480
MD08G1205800.v1.1	-2.543179	0.02111267	- && - && PF14686:Polysaccharide lyase family 4, domain II|PF14683:Polysaccharide lyase family 4, domain III|PF06045:Rhamnogalacturonate lyase family	Chr08	26640920	26645855	-	1923
MD02G1094400.v1.1	-1.158766	0.021492098	- && - && PF07911:Protein of unknown function (DUF1677)	Chr02	7491531	7491980	-	450
MD07G1216200.v1.1	-1.231445	0.021901051	- && P32068.1 RecName: Full=Anthranilate synthase alpha subunit 1, chloroplastic; AltName: Full=Anthranilate synthase component 1-1; AltName: Full=Anthranilate synthase component I-1; AltName: Full=Protein A-METHYL TRYPTOPHAN RESISTANT 1; AltName: Full=Protein JASMONATE-INDUCED DEFECTIVE LATERAL ROOT 1; AltName: Full=Protein TRYPTOPHAN BIOSYNTHESIS 5; AltName: Full=Protein WEAK ETHYLENE INSENSITIVE 2; Flags: Precursor && PF04715:Anthranilate synthase component I, N terminal region|PF00425:chorismate binding enzyme	Chr07	29367302	29371681	+	1731
MD15G1360600.v1.1	-1.20598	0.022220596	- && Q93ZE2.1 RecName: Full=Transcription factor TGA7; AltName: Full=bZIP transcription factor 50; Short=AtbZIP50 && PF00170:bZIP transcription factor|PF14144:Seed dormancy control	Chr15	43422393	43425706	-	1053
novel.1538	-3.007599	0.022721687	-	Chr06	29634076	29635236	+	790
novel.2517	-3.17127	0.02275261	-	Chr10	24872262	24874035	+	1774
MD06G1041000.v1.1	2.1009549	0.024432239	- && Q850K7.2 RecName: Full=Expansin-like B1; AltName: Full=Expansin-related 1; AltName: Full=OsEXLB1; AltName: Full=OsEXPR1; AltName: Full=OsaEXPb3.1; Flags: Precursor && PF01357:Pollen allergen|PF03330:Rare lipoprotein A (RlpA)-like double-psi beta-barrel	Chr06	5217113	5219045	-	768
MD13G1084900.v1.1	-1.055492	0.026083999	- && O04536.1 RecName: Full=Probable galacturonosyltransferase-like 9; AltName: Full=Like glycosyl transferase 8 && -	Chr13	5962525	5964111	-	180
MD01G1212000.v1.1	-3.033771	0.026922375	- && - && -	Chr01	30542233	30553608	+	219
MD17G1202200.v1.1	-1.148377	0.027719758	- && Q9XI23.1 RecName: Full=Boron transporter 4 && PF00955:HCO3-transporter family	Chr17	24486036	24490656	+	1989

MD03G1099800.v1.1	-1.917214	0.028162249	- && Q9LX14.1 RecName: Full=Protein UPSTREAM OF FLC && PF06136:Domain of unknown function (DUF966)	Chr03	8524445	8528229	-	1428
MD08G1088700.v1.1	-2.99328	0.028232446	- && - && PF13414:TPR repeat	Chr08	7320617	7322911	-	1899
MD06G1173600.v1.1	-1.678035	0.02896281	- && Q9FM03.2 RecName: Full=Dof zinc finger protein DOF5.6; Short=AtDOF5.6 && PF02701:Dof domain, zinc finger	Chr06	31334478	31336919	-	960
MD14G1055800.v1.1	-1.115475	0.029153415	- && - && PF08879:WRC	Chr14	5700667	5702876	-	1185
MD17G1058800.v1.1	-2.692486	0.029516711	- && Q9LF27.1 RecName: Full=Ribosome biogenesis protein WDR12 homolog; AltName: Full=Pescadillo-interacting protein 2; Short=AtPEIP2 && PF08154:NLE (NUC135) domain	Chr17	4791756	4796215	-	1545
MD14G1036900.v1.1	-1.136859	0.029531028	- && Q9SVD0.1 RecName: Full=Protein SMAX1-LIKE 3; Short=AtSMXL3 && -	Chr14	3386358	3389941	+	2646
novel.2638	-1.76438	0.029719483	PF00628:PHD-finger	Chr10	12436775	12440896	-	1863
MD02G1279500.v1.1	-2.74246	0.030042218	- && - && PF02536:mTERF	Chr02	33598447	33599563	-	567
MD09G1033200.v1.1	-1.249395	0.030672839	- && Q941I0.2 RecName: Full=2-methylene-furan-3-one reductase; AltName: Full=Enone oxidoreductase; Short=FaEO; AltName: Full=Quinone oxidoreductase; Short=FaQR && PF13602:Zinc-binding dehydrogenase|PF08240:Alcohol dehydrogenase GroES-like domain	Chr09	2040090	2041792	-	978
MD07G1146300.v1.1	-1.254042	0.030679431	- && - && -	Chr07	21333862	21336544	+	900
MD05G1026400.v1.1	-1.717489	0.032033909	- && - && -	Chr05	4221021	4222298	+	219
MD00G1015600.v1.1	-1.889636	0.033568566	- && Q50228.1 RecName: Full=Formamidase; AltName: Full=Formamide amidohydrolase && PF03069:Acetamidase/Formamidase family	Chr00	2245255	2247379	-	594
MD05G1281800.v1.1	-1.048941	0.034032647	- && - && PF13855:Leucine rich repeat	Chr05	41568324	41569751	+	1428
MD11G1033300.v1.1	-1.631063	0.034121731	- && Q8RWL6.1 RecName: Full=Serine/threonine-protein kinase STY17; AltName: Full=Serine/threonine/tyrosine-protein kinase 17 && PF07714:Protein tyrosine kinase	Chr11	2883802	2889682	-	960
MD14G1055400.v1.1	-5.699024	0.034526487	- && Q84WD3.2 RecName: Full=Probable disease resistance protein At4g19060 && PF00931:NB-ARC domain	Chr14	5656492	5657150	+	603
MD07G1190600.v1.1	-1.711673	0.034786897	- && - && PF05056:Protein of unknown function (DUF674)	Chr07	27073009	27076299	+	687
MD08G1251000.v1.1	-3.239094	0.034943221	- && Q94KB2.1 RecName: Full=MLO-like protein 13; Short=AtMlo13; Short=AtMlo20 && PF03094:Mlo family	Chr08	31587312	31589699	+	630
MD00G1049100.v1.1	-1.591042	0.035865231	- && Q9SD53.1 RecName: Full=UPF0481 protein At3g47200 && PF03140:Plant protein of unknown function	Chr00	9165843	9167174	-	1332
novel.1533	-1.15746	0.036420405	PF13952:Domain of unknown function (DUF4216)|PF13960:Domain of unknown function (DUF4218)	Chr06	29384468	29386685	+	1859
MD10G1120900.v1.1	-1.144372	0.036420405	- && Q9SKQ4.1 RecName: Full=Pentatricopeptide repeat-containing protein At2g21090 && PF01535:PPR repeat|PF13041:PPR repeat family	Chr10	19769781	19771619	+	1839
MD06G1182800.v1.1	-3.494517	0.037125865	- && Q9XF23.1 RecName: Full=Protein EDS1L; AltName: Full=Enhanced disease susceptibility 1-like && PF01764:Lipase (class 3)	Chr06	32134443	32136908	+	1851
MD10G1160100.v1.1	-1.08384	0.03726239	- && - && -	Chr10	25094282	25097726	+	1647
MD13G1055400.v1.1	-1.314263	0.038278713	- && Q5ZJ01.1 RecName: Full=Protein ABHD17B; AltName: Full=Alpha/beta hydrolase domain-containing protein 17B;	Chr13	3872148	3874390	-	1143

			Short=Abhydrolase domain-containing protein 17B; Flags: Precursor && PF12695:Alpha/beta hydrolase family					
MD07G1036200.v1.1	-4.192578	0.038433891	- && P51074.2 RecName: Full=Annexin-like protein RJ4 && PF00191:Annexin	Chr07	2973537	2977242	-	945
MD09G1249500.v1.1	-2.57391	0.042140569	- && Q9STE1.1 RecName: Full=Pentatricopeptide repeat-containing protein At4g21300 && PF14432:DYW family of nucleic acid deaminases|PF13041:PPR repeat family|PF01535:PPR repeat	Chr09	31877260	31879796	+	1569
MD07G1274100.v1.1	-1.199937	0.042376201	- && Q8LF97.1 RecName: Full=SKP1-like protein 21; Short=AtSK21 && -	Chr07	33967974	33970319	-	210
MD12G1015400.v1.1	1.6311877	0.043652285	- && Q0WTI8.1 RecName: Full=Chaperone protein dnaJ 72; Short=AtDjC72; Short=AtJ72 && PF00226:DnaJ domain	Chr12	1562520	1565798	-	303
MD10G1244400.v1.1	-1.348399	0.044107286	- && O64743.1 RecName: Full=Berberine bridge enzyme-like 15; Short=AtBBE-like 15; AltName: Full=Protein EMBRYO SAC DEVELOPMENT ARREST 28; AltName: Full=Protein MATERNAL EFFECT EMBRYO ARREST 23; Flags: Precursor && -	Chr10	33922952	33925510	-	342
MD10G1217500.v1.1	-1.70008	0.045495744	- && A4VCM0.1 RecName: Full=NAC domain-containing protein 45 && PF02365:No apical meristem (NAM) protein	Chr10	31583926	31588221	-	1209
MD06G1033000.v1.1	-1.25054	0.045634913	- && - && PF02517:CAAX protease self-immunity	Chr06	3956357	3959681	+	897
MD08G1248200.v1.1	-1.387892	0.04578214	- && Q93VV5.1 RecName: Full=Protein NRT1/ PTR FAMILY 4.3; Short=AtNPF4.3; AltName: Full=Nitrate transporter 1.14 && PF00854:POT family	Chr08	31226687	31231330	+	1842
MD11G1275100.v1.1	-3.860336	0.046472243	- && Q944Z5.1 RecName: Full=Probable 2-oxoglutarate-dependent dioxygenase AOP1 && PF03171:2OG-Fe(II) oxygenase superfamily	Chr11	39192608	39193526	+	432
MD10G1020900.v1.1	-1.155241	0.047108905	- && Q40392.1 RecName: Full=TMV resistance protein N && PF00931:NB-ARC domain|PF01582:TIR domain	Chr10	2562756	2569398	+	3534
MD13G1068000.v1.1	-1.297937	0.047909116	- && - && -	Chr13	4668333	4669400	+	1068
novel.913	-1.44193	0.048572596	-	Chr03	37328108	37329419	-	233
MD04G1110900.v1.1	-1.313572	0.049312352	- && - && -	Chr04	19556875	19560849	-	264
MD17G1025600.v1.1	-1.199585	0.049709071	- && Q9FL59.1 RecName: Full=FT-interacting protein 1 && PF00168:C2 domain|PF08372:Plant phosphoribosyltransferase C-terminal	Chr17	1860329	1863094	-	2556

Table S3: Pearson correlation coefficient (upper value) and p-value (lower value) between RNA-Seq and qRT-PCR data for the six genes associated with the jasmonic acid biosynthesis and signaling pathway. Significant association at P-value ≤ 0.05 are highlighted and are assigned with asterisk.

	MdMYC2	*MdMYC4*	*MdJAZ12*	*MdJAZ2*	*MdLOX-2.1*	*MdLOX-5*
MdMYC2	0.8261<br>0.2336					
MdMYC4		0.9144<br>**0.0351****				
MdJAZ12			0.6326<br>0.6774			
MdJAZ2				0.6057<br>**0.0078*****		
MdLOX-2.1					0.9144<br>**0.0003*****	
MdLOX-5						0.1783<br>**5E-7*****
